# Circulating IL-17 reduces the risk of cisplatin-induced hearing loss in children: a bidirectional two-sample Mendelian randomization study

**DOI:** 10.1038/s41598-023-46299-2

**Published:** 2023-11-02

**Authors:** Ying Xu, Caijuan Huang, Jingjing Liu, Yaying Xu, Haiping Yang

**Affiliations:** 1 Department of Hematology, The First Affiliated Hospital, and College of Clinical Medicine of Henan University of Science and Technology, Luoyang, 471003 China; 2 Department of Endocrinology, The First Affiliated Hospital, and College of Clinical Medicine of Henan University of Science and Technology, Luoyang, 471003 China

**Keywords:** Paediatric cancer, Genome-wide association studies

## Abstract

Studies have reported that cytokines and their related signaling pathways play a role in inner ear diseases. In clinical practice, approximately 50% of pediatric cancer patients experience irreversible hearing loss after cisplatin treatment. However, currently, there is a lack of systematic research on the causal relationship between circulating cytokines and cisplatin-induced hearing loss in children. Genetic variant data for 41 circulating cytokines were obtained from a meta-analysis of genome-wide association studies (GWAS) among 8293 individuals of Finnish descent. The GWAS data for Cisplatin-induced hearing loss in children were derived from a multicenter cohort of European pediatric cancer patients and survivors (N = 390), including both cases with hearing loss after cisplatin chemotherapy and controls without hearing loss. Multiple methods were employed for bidirectional Mendelian randomization (MR) estimation. Bonferroni correction was applied to adjust the original P-values, followed by a series of sensitivity analyses. In the directional Mendelian randomization (MR) analysis, it was found that IL-17 was significantly associated with a reduced risk of Cisplatin-induced hearing loss in children (OR: 0.18, CI: 0.06–0.48, P < 0.001, FDR = 0.041). In the reverse MR analysis, there were some nominal causal relationships of Cisplatin-induced hearing loss in children with certain cytokines [M-CSF: (OR: 1.04, CI: 1.01–1.08, P = 0.010, FDR = 0.41); IL-2RA: (OR: 1.03, CI: 1.00–1.05, P = 0.044, FDR = 0.447); MIP-1β: (OR: 1.02, CI: 1.00–1.04, P = 0.041, FDR = 0.447)]. leave-one-out analysis demonstrated that only M-CSF exhibited stability. These findings reveal a causal relationship between IL-17 and cisplatin-induced hearing loss in children. Further research is needed to determine the potential protective mechanisms of IL-17 in cisplatin-induced ototoxicity.

## Introduction

Hearing loss is a multifactorial condition that can manifest across all age groups, with etiological factors varying based on age and environmental influences. Notable contributors to hearing loss include genetic mutations, prolonged exposure to loud noises, and ototoxic pharmaceutical agents^1^. It has been reported that over half a million people suffer permanent hearing loss each year due to the use of therapeutic drugs that have ototoxic side effects. There is an unmet clinical need to prevent such hearing loss without compromising the therapeutic efficacy of these life-saving drugs^2^. Cisplatin is a commonly used ototoxic drug, however, there exists considerable heterogeneity in the extent of hearing loss experienced by patients^3^. Ototoxicity induced by platinum-based drugs may persist throughout a patient's lifetime, yet, thus far, there are no FDA-approved drugs for preventing cisplatin-induced ototoxicity^4^.

Cisplatin has three main targets within the inner ear: hair cells, spiral ganglion neurons, and the vascular stria (cochlear metabolic center)^5^. Studies have indicated that one of the causes of cisplatin-induced ototoxicity is aberrant oxidative stress and excessive accumulation of reactive oxygen species (ROS). Decreasing intracellular ROS levels can protect cells from cisplatin-induced apoptosis^6^. Furthermore, a higher level of autophagy can enhance the resistance of hair cells to cisplatin ototoxicity. For instance, glycogen synthase kinase-3β and regulators such as 3-methyladenine and chloroquine can modulate autophagy levels, thus exerting either protective or detrimental effects on hearing loss^7–9^. Autophagy and oxidative stress are intricate biological processes regulated by various signals and cytokines. However, the association between circulating cytokines and cisplatin-induced ototoxicity remains largely unexplored. Therefore, comprehending the precise role of circulating cytokines and the risk of cisplatin-induced ototoxicity in children may contribute to the identification of potential preventive, predictive, and therapeutic targets.

Mendelian randomization, an increasingly widely used tool in genetic epidemiology, is employed to infer causal relationships reliably and credibly by combining the random assortment of genetic variants related to exposure (such as circulating cytokines) as instrumental variables (IVs) to assess the causal relationship between the exposure and the outcome, enabling inferences about causal relationships regarding the outcome^10,11^. This study aims to analyze the causal relationship between 41 circulating cytokines and cisplatin-induced hearing loss in children using a bidirectional two-sample MR approach.

## Methods

### Study design

**Figure 1:**
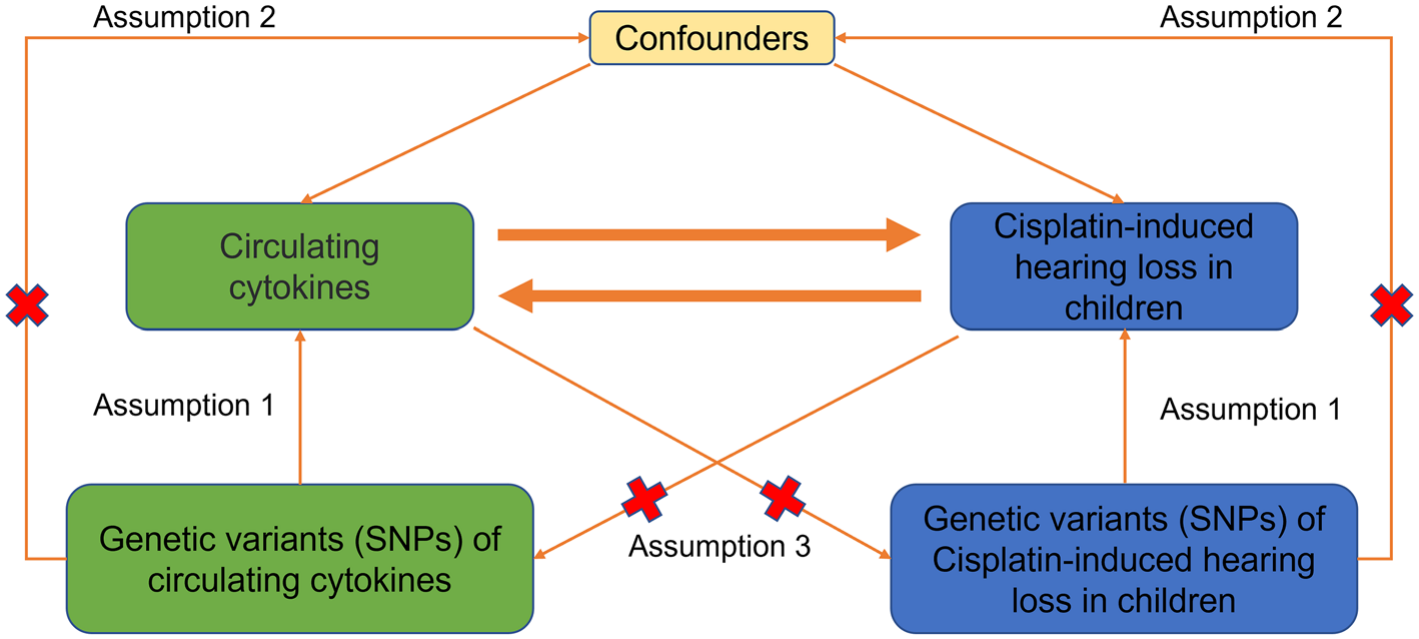
Mendelian randomization study flowchart revealing the causal relationship between circulating cytokines and cisplatin-induced hearing loss in children.

This study utilizes a bidirectional two-sample Mendelian randomization (MR) approach to assess the causal effects between circulating cytokine levels and cisplatin-induced pediatric hearing impairment, aiming to improve the quality of information based on the Strengthening the Reporting of Observational Studies in Epidemiology for Mendelian Randomization (STROBE-MR) guidelines^12,13^. The MR study design workflow is illustrated in Fig. 1. Firstly, MR analysis is conducted to investigate the causal relationship between circulating cytokines and hearing impairment based on the following assumptions: (1) genetic instruments are strongly associated with the exposure, (2) genetic instruments are independent of any potential known confounding factors, and (3) the genetic instrument-outcome association is solely mediated by the exposure. Additionally, a reverse MR analysis is employed to explore potential reverse causal effects. All data utilized in this study are obtained from publicly available large-scale genome-wide association studies (GWAS), each of which has obtained ethical approval from the corresponding ethics committees. Informed consent was also obtained in the original studies.

### Data sources

We selected summary statistics data on the concentrations of 41 circulating cytokines from a comprehensive meta-analysis of the largest and most recent genome-wide association studies (GWAS)^14^. This analysis encompasses 8293 participants from three independent cohort studies: FINRISK 1997, FINRISK 2002, and the Finnish Cardiovascular Risk in Young Finns Study (Supplementary Table 1). The summary statistical data on cisplatin-induced hearing loss in childhood cancer (ID: ebi-a-GCST90013831) in this study were derived from a multicenter cohort of European pediatric cancer patients and survivors. The cohort included children who received treatment with a single platinum-based drug throughout their cancer treatment, and baseline hearing loss was excluded. The cohort consisted of 222 children who experienced hearing loss after cisplatin chemotherapy and 168 children who received cisplatin treatment as controls but did not experience hearing loss. These strict inclusion criteria ensured the credibility and validity of the causal relationship between cisplatin-induced ototoxicity and the use of platinum-based drugs^15^. Specifically, participants were recruited through a network of 14 institutions across 7 countries: Switzerland, Italy, Czech Republic, Denmark, Germany, Austria, and the Netherlands. The inclusion criteria for this cohort were: (1) diagnosis of cancer before the age of 19; (2) initial treatment with cisplatin as a single platinum agent during childhood cancer therapy or a switch from cisplatin to carboplatin during therapy; (3) no cranial or otic radiation therapy; (4) completion of chemotherapy; (5) at least one pure-tone audiometry assessment within 2 years after completion of chemotherapy; (6) availability of biological material (blood or saliva) for DNA extraction; and (7) no baseline hearing loss^16^.

### Instrumental variables

To ensure the validity of the results, the MR analysis was conducted following three quality control steps to identify instrumental variables (IVs): (1) We selected SNPs closely associated with circulating cytokines, with genome-wide significance (P-value < 5 × 10^−6^), as potential IVs. (2) To eliminate linkage disequilibrium (LD), a threshold of LD r^2^ < 0.001 with a window size of 10,000 kb was set, and SNPs with an r^2^ > 0.001 with the most significant SNP within the 10,000 kb range were removed. (3) If no SNPs for the cytokines were available, we searched for proxy SNPs with an LD r^2^ > 0.8. (4) To assess instrument strength, we calculated the F-statistic using the formula: F = (N − 2) × R^2^/(1 − R^2^). F-statistics greater than 10 indicate sufficient instrument strength, while F-statistics below 10 led to exclusion of the SNP from the MR analysis^17^. In the above formula, R^2^ represents the extent to which instrumental variables explain exposure, and N represents the sample size in GWAS analysis of exposure-related SNPs. R^2^ was calculated as follow: R^2^ = 2 × β^2^ × EAF × (1 − EAF)^18^. (5) Palindromic SNPs and SNPs that could not be aligned were removed.

### Statistical analysis

**Figure 2:**
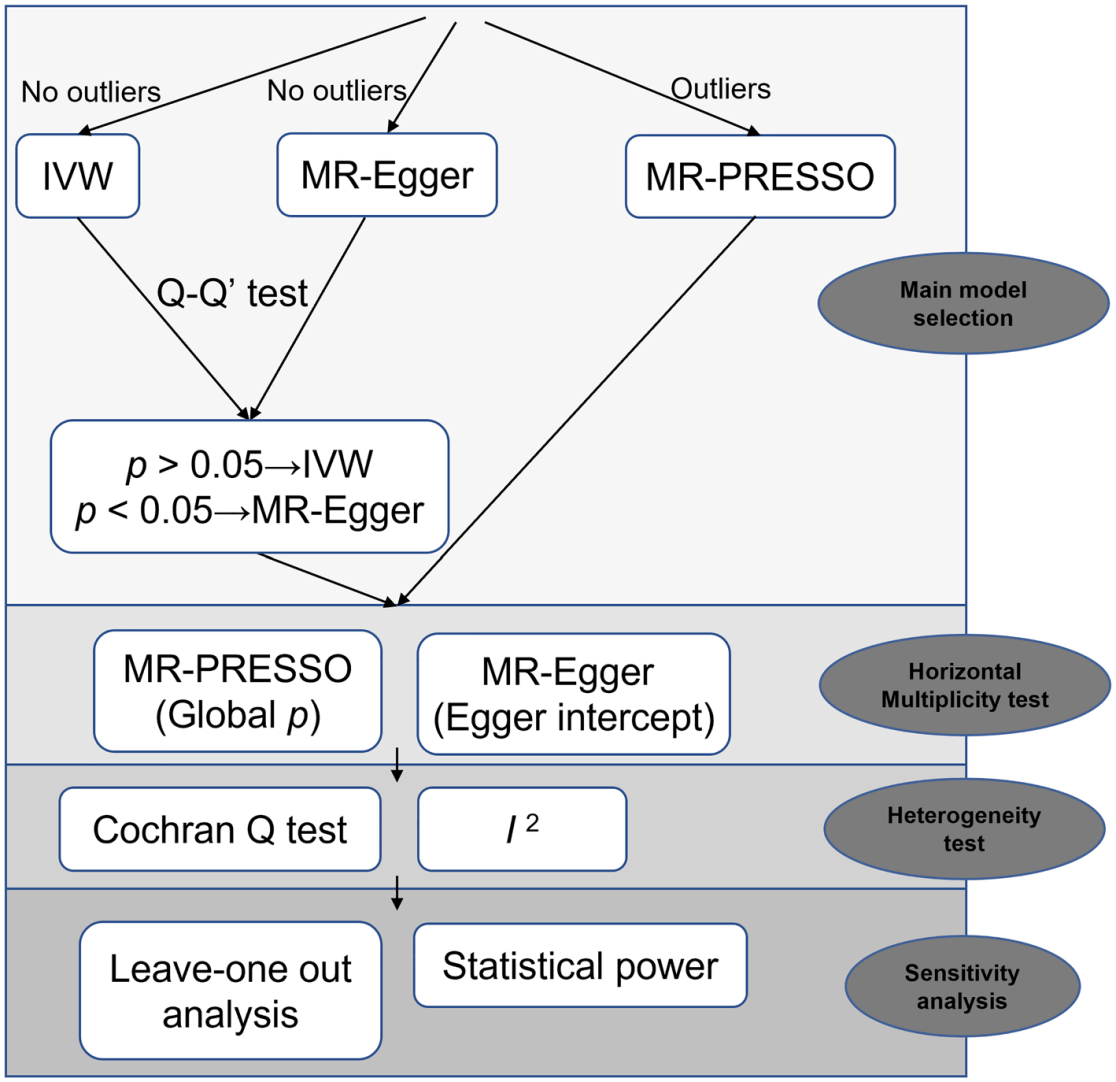
Statistical analysis flowchart revealing the causal relationship between circulating cytokines and cisplatin-induced hearing loss in children.

In MR analysis, the inverse-variance weighted (IVW) method is considered the primary analytical approach, employing a random-effects model to assess the causal relationship between circulating cytokines and Cisplatin-induced hearing loss^19^. However, in the presence of potential pleiotropy or noticeable outliers, we employed MR-pleiotropy residual sum and outlier (MR-PRESSO) as the main analytical method. MR-PRESSO has the capability to detect and correct for potential directional pleiotropy outliers^20^. Additionally, other supplementary MR methods such as MR-Egger regression and Weighted Median were performed to assess the robustness of the results. It should be noted that when MR-PRESSO indicates the absence of outliers, Rucker's Q'-test is used to identify heterogeneity in the MR-Egger model and determine whether IVW or MR-Egger is the most appropriate primary model based on the P value of difference in Q–Q′ values^21^. Cochran's Q test and *I*^2^ statistic were used to assess heterogeneity among different instruments, where *I*^2^ > 25% and a P-value < 0.05 in the Cochran's Q test were considered indicative of potential heterogeneity^22,23^. The P-values for the 41 circulating cytokines were corrected using the false discovery rate(FDR) method, and only those with a FDR < 0.05 were deemed to have relatively convincing causal relationships, while those with FDR > 0.05 and P < 0.05 were considered nominally significant causal relationships^24^. Leave-one-out cross-validation analysis was performed for all nominally causal exposures and outcomes to assess whether the comprehensive IVW estimate was driven by any single SNP. Furthermore, prior statistical power for the association between circulating cytokines and Cisplatin-induced hearing loss was calculated using https://shiny.cnsgenomics.com/mRnd/ with a Type I error rate of 0.05, and statistical power for Cisplatin-induced hearing loss in relation to circulating cytokines was calculated using https://sb452.shinyapps.io/power/ at a significance level of 0.05^25,26^. The analysis workflow is depicted in Fig. 2. All analyses were implemented using R version 4.2.3 (http://www.r-project.org), and a P-value < 0.05 was considered statistically significant.

### Ethical approval

This study utilized published articles or publicly available GWAS summary data. No additional original data collection was performed, thus ethical approval from a medical ethics committee was not required. Each included study had already obtained approval from its respective institutional ethics review board, and all methods were performed in accordance with the relevant guidelines and regulations.

## Results

### Instrumental variables and main explanatory models

Under the relatively lenient threshold of P < 1 × 10^−5^, all 41 cytokines exhibited independent SNPs, and a total of 413 SNPs that met the criteria. At the genome-wide significance level, 3 to 18 independent SNPs were identified as instrumental variables (IVs) for cytokines, with corresponding F-statistics ranging from 14.3 to 957.3 (Supplementary Table 5). A total of 19 genetic instruments associated with cytokines and unavailable in the outcome data were successfully proxyed (Supplementary Table 2). In the reverse MR analysis, 7 SNPs associated with cisplatin-induced hearing loss in children were identified, with F-statistics ranging from 110.7 to 240.6 (Supplementary Table 6). The appropriate model for interpreting causal relationships was determined based on Fig. 2. In the directional MR analysis, there were no outliers detected in the MR-PRESSO results for all 41 cytokines. However, according to the results of the Rucker framework, MR-Egger was suggested as an appropriate model for MCP-1, while IVW was suitable for interpreting the causal relationships of the other 40 cytokines (Supplementary Table 3). In the reverse MR analysis, based on the MR-PRESSO results, rs949800 was excluded from explaining the causal relationship between cisplatin-induced pediatric hearing impairment and IL-18. Similarly, according to the results of the Rucker framework, MR-Egger was suggested as an appropriate model for MCP-3 and SDF-1A, while IVW was suitable for interpreting the causal relationships of the other 38 cytokines (Supplementary Table 4).

### Causal effect of genetically predicted circulating cytokines on cisplatin-induced hearing loss in children

**Figure 3:**
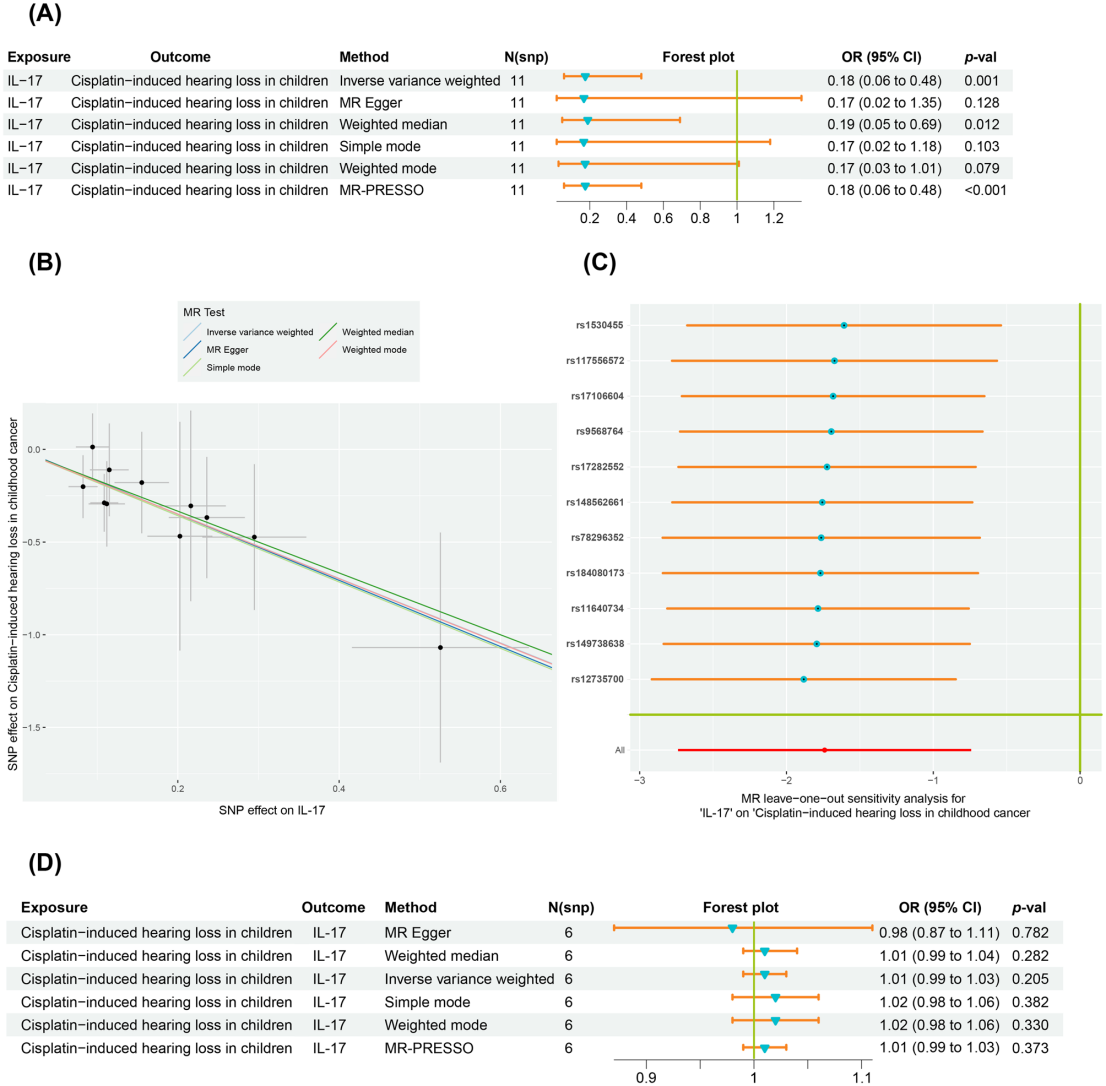
Bidirectional causal relationship between genetically predicted IL17 and cisplatin-induced hearing loss in children. (**A**) Genetically predicted IL17 on the risk of cisplatin-induced hearing loss. (**B**) The scatter plots in relation to IL17 and the associated risk of cisplatin-induced hearing loss in children. (**C**) Leave-one-out analysis of Mendelian randomization (MR) estimates of genetic risk of IL17 on cisplatin-induced hearing loss in children. (**D**) Genetically predicted cisplatin-induced hearing loss on the level of circulating IL17. *IL-17* interleukin-17, *OR* odds ratio, *CI* confidence interval.

After correcting the original P-values using the Bonferroni method, IL17 was found to decrease the risk of cisplatin-induced hearing loss in children (OR: 0.18, CI: 0.06–0.48, P < 0.001, FDR = 0.041) (Fig. 3A,B, Supplementary Table 3). According to the Cochran Q-test and *I*^2^, there was no evidence of heterogeneity in the IVW model (P for Q-statistic = 0.996,* I*^2^ = 0.00%). The MR-Egger intercept revealed no indication of directional pleiotropy (P for Egger intercept = 0.963), and the results of MR-PRESSO also suggested the absence of directional pleiotropy (Global P = 0.997). Furthermore, the leave-one-out analysis confirmed that the pooled IVW estimate was not reliant upon any single SNP (Fig. 3C). As shown in Fig. 3D, reverse MR analysis indicates no significant causal effect of cisplatin-induced hearing loss in children on circulating IL-17.

### Causal effect of genetically predicted cisplatin-induced hearing loss in children on circulating cytokines

**Figure 4:**
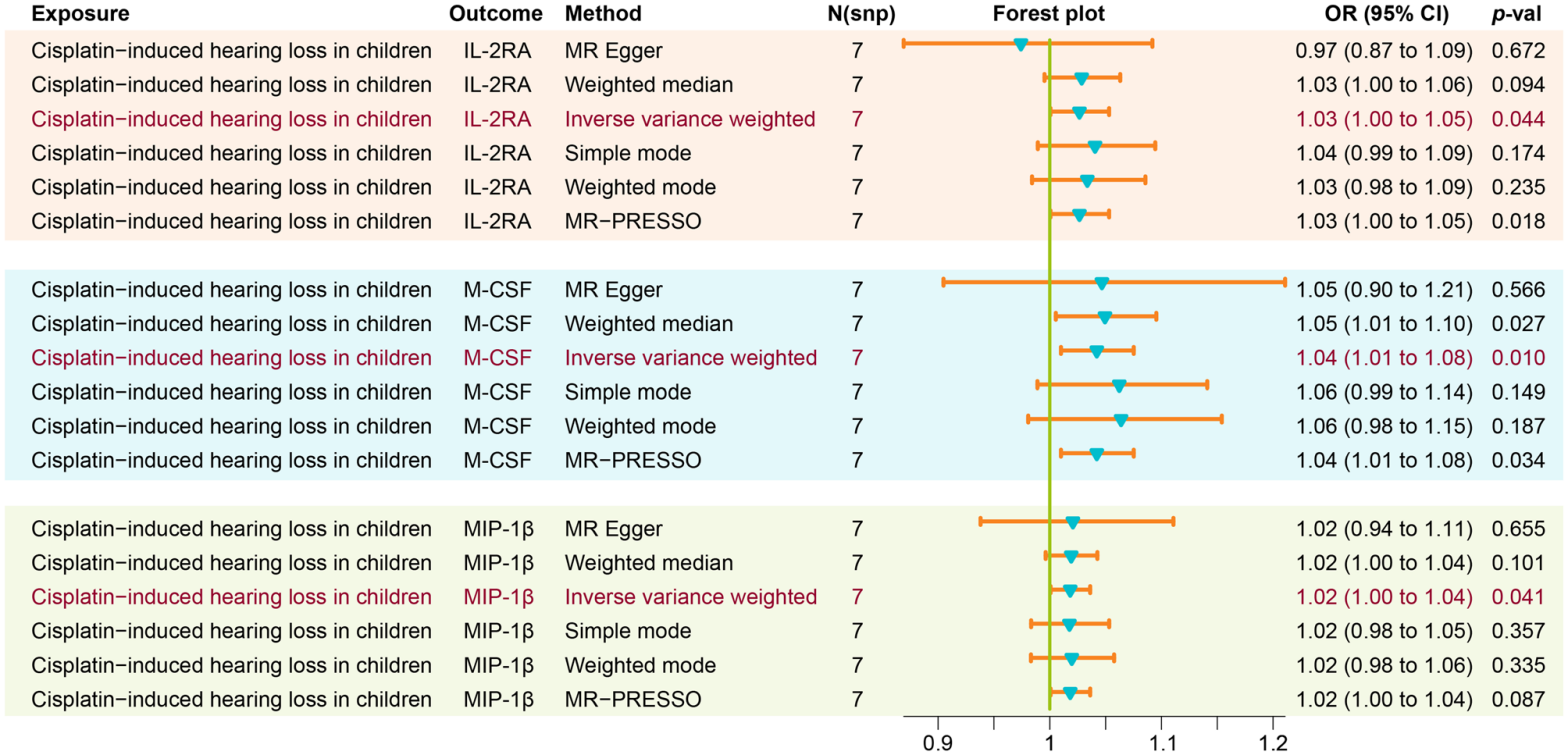
Genetically predicted cisplatin-induced hearing loss on the level of circulating IL-2RA, M-CSF, and MIP-1β. *IL-2RA* Interleukin 2 Receptor Subunit Alpha, *M-CSF* Macrophage colony-stimulating factor, *MIP-1β* Macrophage inflammatory protein-1 beta, *OR* odds ratio, *CI* confidence interval.

**Figure 5:**
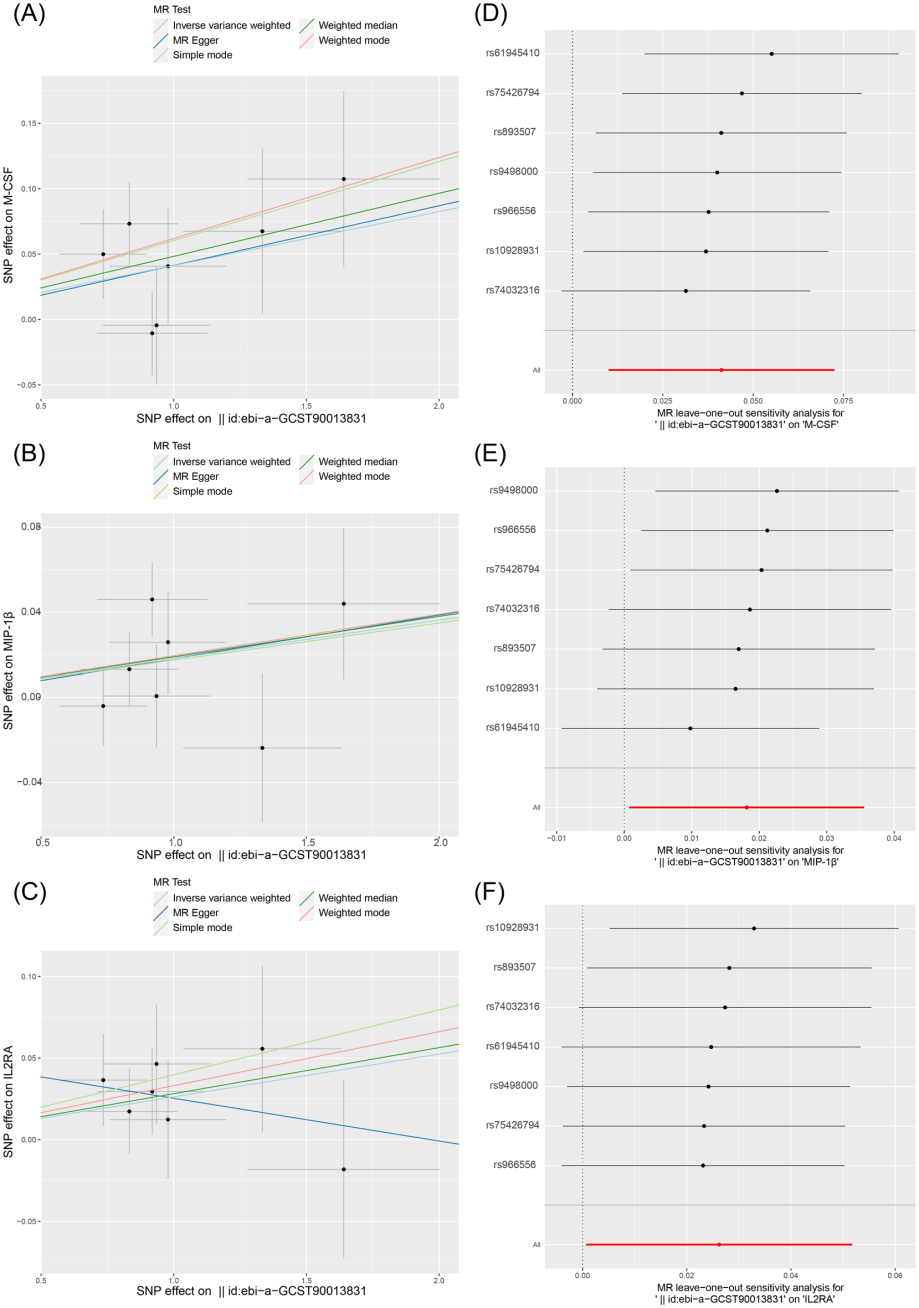
The scatter plots in relation to cisplatin-induced hearing loss in children on the level of (**A**) M-CSF, (**B**) MIP-1β and (**C**) IL-2RA. Leave-one-out analysis of Mendelian randomization (MR) estimates of genetic risk of cisplatin-induced hearing loss in children on the level of (**D**) M-CSF, (**E**) MIP-1β and (**F**) IL-2RA. *IL-2RA* Interleukin 2 Receptor Subunit Alpha, *M-CSF* Macrophage colony-stimulating factor, *MIP-1β* Macrophage inflammatory protein-1 beta.

In the reverse MR analysis, there were nominal causal relationships found between cisplatin-induced hearing loss and cytokines. Cisplatin-induced hearing loss was associated with increased levels of three circulating cytokines: M-CSF (OR: 1.04, 95% CI: 1.01–1.08, P = 0.010, FDR = 0.41); IL-2RA (OR: 1.03, 95% CI: 1.00–1.05, P = 0.044, FDR = 0.447); and MIP-1β (OR: 1.02, 95% CI: 1.00–1.04, P = 0.041, FDR = 0.447) (Fig. 4). However, in the leave-one-out analysis, only the result for M-CSF remained relatively robust (Fig. 5). No causal relationship was observed between genetically predicted cisplatin-induced hearing loss and the levels of 38 other circulating cytokines (Supplementary Table 4).

## Discussion

To the best of our knowledge, the relationship between cytokines and cisplatin-induced ototoxicity remains unclear. By implementing this MR analysis, we were able to surpass limitations and establish a more robust research design, allowing us to confidently investigate the causal relationship. This is the first comprehensive bidirectional MR study aimed at investigating the association between genetically determined Circulating cytokines and cisplatin-induced ototoxicity. Through analyzing 41 cytokines using the largest available GWAS dataset, we identified a potential causal relationship between IL-17 and cisplatin-induced ototoxicity.

Approximately one-third of childhood cancer survivors (CCS) suffer from severe, disabling, or life-threatening chronic conditions due to cancer or its treatments^27,28^. For CCS who have received platinum-based chemotherapy or cranial radiation therapy, this may include the risk of treatment-induced hearing loss^29–32^. A Cochrane review in 2016 found that 1.7% to 90.1% of chronic disease patients experienced some degree of hearing loss after receiving platinum-based chemotherapy. As CCS often experience hearing loss during critical stages of childhood development, they may be susceptible to lifelong impacts on quality of life^33–35^. Cisplatin induces damage to inner ear cells, including cochlear hair cells, by inducing cell apoptosis, primarily through the excessive generation and accumulation of reactive oxygen species (ROS) within the cells^36^. Numerous cytokines are involved in the process of apoptosis induction. There is also evidence that cisplatin-related ototoxicity has a genetic susceptibility. Tserga et al.^37^ identified eight different SNPs from five different genes that showed significant correlations with cisplatin ototoxicity in multiple studies. These genes are predominantly related to antioxidant regulation, neurotransmission, or auditory function, providing genetic evidence for the ototoxic mechanisms of cisplatin. However, the causal relationship between genetic-level cytokines and cisplatin-induced ototoxicity is currently lacking. In this study, by screening 41 cytokines in the largest GWAS dataset, we observed a bidirectional relationship between cytokines and cisplatin-induced ototoxicity that can predict each other. On the one hand, IL17 was found to reduce the risk of cisplatin-induced hearing loss, and this causal relationship remained significant even after Bonferroni correction. On the other hand, cisplatin-induced hearing loss was associated with a nominal increase in M-CSF levels.

Upregulation of autophagy levels by IL-17 may be a molecular mechanism by which it reduces the risk of cisplatin-induced ototoxicity. Studies have shown that the presence of autophagosomes and higher levels of autophagy gene expression are associated with more intact morphology and less severe damage to cochlear hair cells^9^. Inhibition of glycogen synthase kinase-3β (GSK-3β), a downstream factor of AKT, which acts as an inhibitor of autophagy, can promote the survival of cochlear cells by selectively upregulating autophagy. Conversely, autophagy inhibitors such as chloroquine may exacerbate the severity of cisplatin-induced hearing loss^7,8^. In addition, PRDX1 activates autophagy through the PTEN-AKT signaling pathway, thereby preventing cisplatin-induced damage to spiral ganglion neurons^38^. Not only that, Trehalose has been reported to prevent cisplatin-induced damage to cochlear hair cells by activating TFEB-mediated autophagy^39^. IL-17 has been shown to promote the expression of key autophagy proteins such as Beclin-1 and LC3^40–45^. Importantly, IL-17/IL-17R-induced autophagy has been shown to confer resistance to oxaliplatin in hepatocellular carcinoma (HCC), and a similar mechanism may exist in protecting cochlear hair cells against cisplatin-induced damage^46^. Additionally, IL-17 may be crucial for the development of cisplatin resistance in several major cancers^47,48^. Based on the findings of this study, IL-17 could potentially elevate the autophagy level in hair cells, leading to a reduction in cisplatin-induced ototoxicity. However, further investigation is still needed to substantiate this claim.

In a reverse causal relationship, there is a nominal causal association between cisplatin-induced ototoxicity and circulating M-CSF levels. Macrophage colony-stimulating factor (M-CSF), also known as colony-stimulating factor 1 (CSF1), is a cytokine secreted by osteoblasts. It is a hematopoietic growth factor involved in the proliferation, differentiation, and survival of monocytes, macrophages, and bone marrow progenitor cells. Studies have demonstrated that Granulocyte colony-stimulating factor (G-CSF) mobilized bone marrow cells can prevent cisplatin-induced tubular damage, and M-CSF synergistically interacts with G-CSF^49^. Additionally, M-CSF enhances platelet recovery following cisplatin/carboplatin chemotherapy in ovarian cancer patients^50^. Therefore, it is hypothesized that an increase in circulating M-CSF levels may be associated with the resistance of body to cisplatin-induced bone marrow and kidney toxicity.

This study has several advantages. Firstly, in the MR analysis, the summary data of 41 Circulating cytokines used were derived from the latest and largest-scale GWAS, which enhances the stability and accuracy of effect estimation. Secondly, two-sample bidirectional Mendelian randomization simulates a natural randomized controlled trial by randomly allocating genetic variations, effectively minimizing common confounding factors and helping to distinguish the direction of causality. Thirdly, we combined the statistical results from three models, namely IVW, MR-Egger, and MR-PRESSO, to select the best causal inference model. The directionality of the beta coefficient was assessed using the dual test of MR-PRESSO and Egger-intercept. It is crucial to emphasize the importance of consistent beta directionality across different models in MR analysis, and our results are consistent with this principle^51^. Fourthly, conclusions were obtained based on Bonferroni correction in this study. Bonferroni correction is a conservative method that ensures control over the error rate, providing reliability and robustness to the research findings. Lastly, the original cohort of GWAS for cisplatin-induced hearing loss that we selected consisted of children treated with a single platinum-based drug throughout their cancer treatment, thereby largely excluding confounding caused by cranial or inner ear radiation.

However, our study also has some limitations. Firstly, although the cohort used in the GWAS for cisplatin -induced hearing loss represents the largest to date in terms of non-cranial irradiation and platinum-treated pediatric cancer patients and survivors, the sample size is still relatively small, which could lead to underpowered and unstable results^16^. Secondly, the limited number of significant SNPs obtained using a stricter P-value threshold (P < 5 × 10^−8^) may hinder further investigation. Therefore, we adopted a cutoff of P < 1 × 10^−5^, which may increase the risk of type I errors and insufficient statistical power. We addressed this issue through subsequent sensitivity analyses and F-statistics. Thirdly, ideally, in causal inference, the exposure should precede the outcome. In this study, while germline variants are determined at birth, the temporal relationship between circulating cytokines and hearing loss is less clear. In this regard, the study assumes that the genetic variants selected are good proxies for lifelong cytokine levels. However, actual cytokine levels can be influenced by various factors over time, such as age. Unfortunately, there were no age-stratified GWAS data relevant to this study, which limited further analysis. Fourthly, the biological functions of the selected SNPs are still unknown, making it difficult to completely rule out pleiotropy. However, it is reassuring that the effect estimates were robust across different MR models, and our latest sensitivity analysis results based on various assumptions did not detect any level of pleiotropy in our study. Fifthly, the statistical power of our MR analysis may currently be insufficient. As shown in Supplementary Table 3, the statistical power for most cytokines as exposures was less than 0.8, but it is noteworthy that the statistical power for IL-17 was 1, which enhances the reliability and interpretability of the findings in this study. Finally, these findings have not been validated in clinical and basic research. Therefore, caution should be exercised in interpreting potential causal associations, and further studies are still needed to investigate the underlying mechanisms.

## Conclusion

In conclusion, our findings reveal a causal relationship between IL-17 and cisplatin-induced hearing loss in children. Further research is needed to determine the potential protective mechanisms of IL-17 in cisplatin-induced ototoxicity.

### Supplementary Information

Supplementary Tables.

## Data Availability

All data used in the study were taken from published articles or publicly available GWAS platforms, and all data were freely available.

## References

[1] Nieman CL, Oh ES (2020). Hearing loss. Ann. Intern. Med..

[2] Lee J, Fernandez K, Cunningham LL (2023). Hear and now: Ongoing clinical trials to prevent drug-induced hearing loss. Annu. Rev. Pharmacol. Toxicol..

[3] Skinner R, Pearson AD, Amineddine HA, Mathias DB, Craft AW (1990). Ototoxicity of cisplatinum in children and adolescents. Br. J. Cancer.

[4] Rabiço-Costa D, Gil-da-Costa MJ, Barbosa JP, Bom-Sucesso M, Spratley J (2020). Platinum-drugs ototoxicity in pediatric patients with brain tumors: A 10-year review. J. Pediatr. Hematol. Oncol..

[5] van Ruijven MW, de Groot JC, Klis SF, Smoorenburg GF (2005). The cochlear targets of cisplatin: An electrophysiological and morphological time-sequence study. Hear. Res..

[6] Mukherjea D (2006). Expression of the kidney injury molecule 1 in the rat cochlea and induction by cisplatin. Neuroscience.

[7] Liu T (2019). Enhancing autophagy by down-regulating GSK-3β alleviates cisplatin-induced ototoxicity in vivo and in vitro. Toxicol. Lett..

[8] Liang Z (2021). Metformin alleviates cisplatin-induced ototoxicity by autophagy induction possibly via the AMPK/FOXO3a pathway. J. Neurophysiol..

[9] Jing-Chun H (2011). Modulation of copper transporters in protection against cisplatin-induced cochlear hair cell damage. J. Otol..

[10] Emdin CA, Khera AV, Kathiresan S (2017). Mendelian randomization. JAMA.

[11] Lawlor DA, Harbord RM, Sterne JA, Timpson N, Davey Smith G (2008). Mendelian randomization: Using genes as instruments for making causal inferences in epidemiology. Stat. Med..

[12] Skrivankova VW (2021). Strengthening the reporting of observational studies in epidemiology using Mendelian randomization: The STROBE-MR statement. JAMA.

[13] Skrivankova VW (2021). Strengthening the reporting of observational studies in epidemiology using mendelian randomisation (STROBE-MR): Explanation and elaboration. BMJ.

[14] Ahola-Olli AV (2017). Genome-wide association study identifies 27 loci influencing concentrations of circulating cytokines and growth factors. Am. J. Hum. Genet..

[15] Clemens E (2019). Genetic determinants of ototoxicity during and after childhood cancer treatment: Protocol for the PanCareLIFE study. JMIR Res. Protoc..

[16] Meijer AJM (2021). TCERG1L allelic variation is associated with cisplatin-induced hearing loss in childhood cancer, a PanCareLIFE study. NPJ Precis. Oncol..

[17] Pierce BL, Ahsan H, Vanderweele TJ (2011). Power and instrument strength requirements for Mendelian randomization studies using multiple genetic variants. Int. J. Epidemiol..

[18] Liu Z (2022). Dissecting causal relationships between nonalcoholic fatty liver disease proxied by chronically elevated alanine transaminase levels and 34 extrahepatic diseases. Metabolism.

[19] Burgess S, Butterworth A, Thompson SG (2013). Mendelian randomization analysis with multiple genetic variants using summarized data. Genet. Epidemiol..

[20] Verbanck M, Chen CY, Neale B, Do R (2018). Detection of widespread horizontal pleiotropy in causal relationships inferred from Mendelian randomization between complex traits and diseases. Nat. Genet..

[21] Bowden J (2017). A framework for the investigation of pleiotropy in two-sample summary data Mendelian randomization. Stat. Med..

[22] Greco MF, Minelli C, Sheehan NA, Thompson JR (2015). Detecting pleiotropy in Mendelian randomisation studies with summary data and a continuous outcome. Stat. Med..

[23] Bowden J (2019). Improving the accuracy of two-sample summary-data Mendelian randomization: Moving beyond the NOME assumption. Int. J. Epidemiol..

[24] Wu F, Huang Y, Hu J, Shao Z (2020). Mendelian randomization study of inflammatory bowel disease and bone mineral density. BMC Med..

[25] Deng L, Zhang H, Yu K (2020). Power calculation for the general two-sample Mendelian randomization analysis. Genet. Epidemiol..

[26] Burgess S (2014). Sample size and power calculations in Mendelian randomization with a single instrumental variable and a binary outcome. Int. J. Epidemiol..

[27] Landier, W., Hawkins, S. & Leonard, M. Establishing and enhancing services for childhood cancer survivors: Long-term follow-up program resource guide. *Children’s Oncology Group* (2007).

[28] Gibson TM (2018). Temporal patterns in the risk of chronic health conditions in survivors of childhood cancer diagnosed 1970–99: A report from the Childhood Cancer Survivor Study cohort. Lancet Oncol..

[29] Khan A (2018). Hearing loss in adult survivors of childhood cancer treated with radiotherapy. Children (Basel).

[30] Bass JK (2016). Hearing loss in patients who received cranial radiation therapy for childhood cancer. J. Clin. Oncol..

[31] Clemens E (2019). Recommendations for ototoxicity surveillance for childhood, adolescent, and young adult cancer survivors: A report from the International Late Effects of Childhood Cancer Guideline Harmonization Group in collaboration with the PanCare Consortium. Lancet Oncol..

[32] Beyea JA (2020). Long-term incidence and predictors of significant hearing loss requiring hearing assistive devices among childhood cancer survivors: A population-based study. J. Clin. Oncol..

[33] Klassen AF, Anthony SJ, Khan A, Sung L, Klaassen R (2011). Identifying determinants of quality of life of children with cancer and childhood cancer survivors: A systematic review. Support Care Cancer.

[34] Pearson SE, Taylor J, Patel P, Baguley DM (2019). Cancer survivors treated with platinum-based chemotherapy affected by ototoxicity and the impact on quality of life: A narrative synthesis systematic review. Int. J. Audiol..

[35] Roland L (2016). Quality of life in children with hearing impairment: Systematic review and meta-analysis. Otolaryngol. Head Neck Surg..

[36] Wang J (2004). Caspase inhibitors, but not c-Jun NH2-terminal kinase inhibitor treatment, prevent cisplatin-induced hearing loss. Cancer Res..

[37] Tserga E (2019). The genetic vulnerability to cisplatin ototoxicity: A systematic review. Sci. Rep..

[38] Liu W (2021). PRDX1 activates autophagy via the PTEN-AKT signaling pathway to protect against cisplatin-induced spiral ganglion neuron damage. Autophagy.

[39] Li Z (2022). Trehalose protects against cisplatin-induced cochlear hair cell damage by activating TFEB-mediated autophagy. Biochem. Pharmacol..

[40] Shen Y, Wang Z, Tan J, Zhong J, Chen L (2021). TRAF6/ERK/p38 pathway is involved in interleukin-17-mediated autophagy to promote osteoclast precursor cell differentiation. Zhejiang Da Xue Xue Bao Yi Xue Ban.

[41] Zhong J, Wang Z, Yuan W, Shen Y, Chen L (2022). Interleukin-17 promotes osteoclastogenesis and periodontal damage via autophagy in vitro and in vivo. Int. Immunopharmacol..

[42] Ramakrishnan RK (2020). IL-17 induced autophagy regulates mitochondrial dysfunction and fibrosis in severe asthmatic bronchial fibroblasts. Front. Immunol..

[43] Orosz L, Papanicolaou EG, Seprényi G, Megyeri K (2016). IL-17A and IL-17F induce autophagy in RAW 264.7 macrophages. Biomed. Pharmacother..

[44] Bie Q (2021). IL-17B/IL-17RB signaling cascade contributes to self-renewal and tumorigenesis of cancer stem cells by regulating Beclin-1 ubiquitination. Oncogene.

[45] Kim KH (2021). IL-17 deficiency aggravates the streptozotocin-induced diabetic nephropathy through the reduction of autophagosome formation in mice. Mol. Med..

[46] Wu J (2017). Autophagy impacts on oxaliplatin-induced hepatocarcinoma apoptosis via the IL-17/IL-17R-JAK2/STAT3 signaling pathway. Oncol. Lett..

[47] Sui G, Qiu Y, Yu H, Kong Q, Zhen B (2019). Interleukin-17 promotes the development of cisplatin resistance in colorectal cancer. Oncol. Lett..

[48] Fan LL, Xue XZ, Jiao N (2016). In vitro effect of IL-17D on human ovarian carcinoma cells and inherent immunity. J. Biol. Regul. Homeost. Agents.

[49] Iwasaki M (2005). Mobilization of bone marrow cells by G-CSF rescues mice from cisplatin-induced renal failure, and M-CSF enhances the effects of G-CSF. J. Am. Soc. Nephrol..

[50] Suzuki M, Ohwada M, Aida I, Sato I, Tamada T (1994). Macrophage colony-stimulating factor enhances platelet recovery following cisplatin/carboplatin chemotherapy in ovarian cancer. Gynecol. Oncol..

[51] Chen X (2022). Causal relationship between physical activity, leisure sedentary behaviors and COVID-19 risk: A Mendelian randomization study. J. Transl. Med..

