# Supplementary material for: Circulating IL-17 reduces the risk of cisplatin-induced hearing loss in children: a bidirectional two-sample Mendelian randomization study
Source: Sci Rep. 2023 Nov 2;13:18957. doi: 10.1038/s41598-023-46299-2 (PMC10622445; doi:10.1038/s41598-023-46299-2)
Supplement: Supplementary file 1 — Supplementary Tables. [file 41598_2023_46299_MOESM1_ESM.docx]

## Supplementary Table 1 Detailed information regarding studies and datasets used in the present study.

| Variable | Ancestry | Numbers of subjects | Consortium |
| --- | --- | --- | --- |
| B-NGF | European | 3570 | FINRISK 2002, and Young Finns Study |
| CTACK | European | 3670 | FINRISK 2002, and Young Finns Study |
| EOTAXIN | European | 8197 | FINRISK 1997, FINRISK 2002, and Young Finns Study |
| FGF-BASIC | European | 7609 | FINRISK 1997, FINRISK 2002, and Young Finns Study |
| G-CSF | European | 7948 | FINRISK 1997, FINRISK 2002, and Young Finns Study |
| GROA | European | 3544 | FINRISK 2002, and Young Finns Study |
| HGF | European | 8336 | FINRISK 1997, FINRISK 2002, and Young Finns Study |
| IFN-G | European | 7745 | FINRISK 1997, FINRISK 2002, and Young Finns Study |
| IL-10 | European | 7724 | FINRISK 1997, FINRISK 2002, and Young Finns Study |
| IL-12 | European | 8314 | FINRISK 1997, FINRISK 2002, and Young Finns Study |
| IL-13 | European | 3596 | FINRISK 2002, and Young Finns Study |
| IL-16 | European | 3521 | FINRISK 2002, and Young Finns Study |
| IL-17 | European | 7804 | FINRISK 1997, FINRISK 2002, and Young Finns Study |
| IL-18 | European | 3675 | FINRISK 2002, and Young Finns Study |
| IL-1B | European | 3348 | FINRISK 2002, and Young Finns Study |
| IL-1RA | European | 3677 | FINRISK 2002, and Young Finns Study |
| IL-2 | European | 3514 | FINRISK 2002, and Young Finns Study |
| IL-2RA | European | 3716 | FINRISK 2002, and Young Finns Study |
| IL-4 | European | 8168 | FINRISK 1997, FINRISK 2002, and Young Finns Study |
| IL-5 | European | 3403 | FINRISK 2002, and Young Finns Study |
| IL-6 | European | 8233 | FINRISK 2002, and Young Finns Study |
| IL-7 | European | 3448 | FINRISK 2002, and Young Finns Study |
| IL-8 | European | 3565 | FINRISK 2002, and Young Finns Study |
| IL-9 | European | 3673 | FINRISK 2002, and Young Finns Study |
| IP-10 | European | 3724 | FINRISK 2002, and Young Finns Study |
| M-CSF | European | 2498 | FINRISK 1997, FINRISK 2002, and Young Finns Study |
| MCP-1 | European | 8337 | FINRISK 1997, FINRISK 2002, and Young Finns Study |
| MCP-3 | European | 1099 | FINRISK 2002, and Young Finns Study |
| MIF | European | 3533 | FINRISK 2002, and Young Finns Study |
| MIG | European | 3724 | FINRISK 2002, and Young Finns Study |
| MIP-1A | European | 3561 | FINRISK 2002, and Young Finns Study |
| MIP-1B | European | 8287 | FINRISK 1997, FINRISK 2002, and Young Finns Study |
| PDGF-BB | European | 8337 | FINRISK 1997, FINRISK 2002, and Young Finns Study |
| RANTES | European | 3454 | FINRISK 2002, and Young Finns Study |
| SCF | European | 8334 | FINRISK 1997, FINRISK 2002, and Young Finns Study |
| SCGF-B | European | 3721 | FINRISK 2002, and Young Finns Study |
| SDF-1A | European | 7829 | FINRISK 2002, and Young Finns Study |
| TNF-A | European | 3493 | FINRISK 1997, FINRISK 2002, and Young Finns Study |
| TNF-B | European | 1566 | FINRISK 1997, FINRISK 2002, and Young Finns Study |
| TRAIL | European | 8230 | FINRISK 1997, FINRISK 2002, and Young Finns Study |
| VEGF | European | 7162 | FINRISK 1997, FINRISK 2002, and Young Finns Study |
| CIsplatin-induced hearing loss in children | European | 390 | Childhood cancer patients from the PCL consortium |

## Supplementary Table 2 Detailed information of the 19 proxy SNPs

| Exposure | Target-snp | Proxy-snp | Target-a1 | Target-a2 | Proxy-a1 | Proxy-a2 | Beta | SE | EAF | p-val |
| --- | --- | --- | --- | --- | --- | --- | --- | --- | --- | --- |
| EOTAXIN | rs80341932 | rs9319945 | G | A | A | G | 0.101 | 0.0204 | 0.8094 | 7.40E-07 |
| GROA | rs114991247 | rs28421666 | C | T | G | A | -0.2202 | 0.0463 | 0.9226 | 1.97E-06 |
| HGF | rs57146176 | rs4417380 | G | A | C | T | -0.0987 | 0.0208 | 0.7998 | 2.18E-06 |
| IL-16 | rs9706053 | rs61409219 | T | C | C | T | 0.4412 | 0.0928 | 0.0173 | 1.98E-06 |
| IL-17 | rs184080173 | rs78402218 | C | T | A | G | 0.236 | 0.0471 | 0.9693 | 5.39E-07 |
| IL-18 | rs10414578 | rs10409826 | T | C | A | G | -0.1817 | 0.0347 | 0.1295 | 1.64E-07 |
| IL-1RA | rs3876037 | rs5757862 | A | G | G | A | 0.1234 | 0.027 | 0.6783 | 4.73E-06 |
| IL-1RA | rs56134659 | rs7373404 | G | A | C | T | -0.1109 | 0.0236 | 0.4835 | 2.56E-06 |
| IL-7 | rs218247 | rs170121 | C | G | C | T | -0.1343 | 0.0285 | 0.2258 | 2.42E-06 |
| IL-8 | rs183628733 | rs77320742 | C | T | A | G | 0.6547 | 0.1417 | 0.9917 | 3.82E-06 |
| IL-9 | rs4880409 | rs4880410 | C | T | T | C | -0.3552 | 0.0716 | 0.9664 | 6.95E-07 |
| MCP-1 | rs7517040 | rs2518526 | A | G | G | A | -0.097 | 0.019 | 0.2334 | 3.41E-07 |
| MIG | rs112861654 | rs140643750 | G | A | G | A | -0.2682 | 0.0527 | 0.9454 | 3.64E-07 |
| MIG | rs191555775 | rs41272086 | T | A | A | G | 0.2279 | 0.0412 | 0.909 | 3.28E-08 |
| MIG | rs55876513 | rs10029157 | G | T | A | G | 0.1638 | 0.0254 | 0.7131 | 1.05E-10 |
| MIP-1A | rs184154340 | rs117839469 | A | G | A | T | 0.3251 | 0.0689 | 0.0304 | 2.40E-06 |
| MIP-1B | rs5743614 | rs9306967 | T | C | C | G | 0.1115 | 0.0232 | 0.1278 | 1.57E-06 |
| PDGF-BB | rs55680718 | rs17794132 | T | C | A | G | -0.1359 | 0.0245 | 0.1173 | 2.96E-08 |
| SDF-1A | rs10474392 | rs6881171 | A | G | T | A | 0.0934 | 0.0177 | 0.334 | 1.38E-07 |

## Supplementary Table 3 Causal effect of genetically predicted circulating cytokines on cisplatin-induced hearing loss in children

## Supplementary Table 4 Causal effect of genetically predicted cisplatin-induced hearing loss in children on circulating cytokines

## Supplementary Table 5 Detailed information for circulating cytokines-associated SNPs with the risk of cisplatin-induced hearing loss in children

| Exposure | SNP | Alt | Ref | Beta | SE | EAF | p-val | F | R^2^ |
| --- | --- | --- | --- | --- | --- | --- | --- | --- | --- |
| B-NGF | rs28637706 | T | G | -0.1554 | 0.0261 | 0.314 | 2.72E-09 | 37.51047 | 0.010404 |
| B-NGF | rs71641308 | T | C | 0.1969 | 0.0429 | 0.0995 | 4.42E-06 | 24.96209 | 0.006947 |
| B-NGF | rs73472576 | T | C | -0.1146 | 0.0251 | 0.457 | 4.81E-06 | 23.40885 | 0.006518 |
| B-NGF | rs7970581 | T | G | 0.1358 | 0.028 | 0.2662 | 1.22E-06 | 25.89288 | 0.007205 |
| CTACK | rs116303454 | A | G | 0.3754 | 0.081 | 0.0218 | 3.58E-06 | 22.17942 | 0.00601 |
| CTACK | rs118084576 | A | G | 0.5675 | 0.1226 | 0.0112 | 3.66E-06 | 26.35279 | 0.007133 |
| CTACK | rs135564 | A | G | -0.1672 | 0.0267 | 0.2632 | 3.59E-10 | 40.20703 | 0.010843 |
| CTACK | rs141331414 | A | G | 0.1977 | 0.0415 | 0.0914 | 1.89E-06 | 23.96736 | 0.006492 |
| CTACK | rs2070074 | A | G | 0.4401 | 0.0372 | 0.891 | 2.60E-32 | 143.3905 | 0.037622 |
| CTACK | rs55764737 | T | C | 0.5424 | 0.0967 | 0.9846 | 2.01E-08 | 33.01956 | 0.008922 |
| CTACK | rs57338032 | A | G | 0.1443 | 0.0316 | 0.8287 | 4.83E-06 | 21.81332 | 0.005912 |
| CTACK | rs57789542 | T | C | -0.7687 | 0.1659 | 0.9951 | 3.58E-06 | 21.25914 | 0.005762 |
| CTACK | rs60247384 | T | C | 0.1128 | 0.0245 | 0.3512 | 4.30E-06 | 21.39284 | 0.005798 |
| CTACK | rs62578137 | T | C | -0.1311 | 0.0286 | 0.2421 | 4.66E-06 | 23.28195 | 0.006307 |
| CTACK | rs72729450 | T | C | -0.5123 | 0.1094 | 0.0123 | 2.81E-06 | 23.54055 | 0.006377 |
| CTACK | rs7333764 | T | C | 0.2811 | 0.0591 | 0.0412 | 2.00E-06 | 23.04231 | 0.006243 |
| CTACK | rs76395525 | A | G | 0.5193 | 0.1081 | 0.0118 | 1.55E-06 | 23.21469 | 0.006289 |
| EOTAXIN | rs11087905 | A | C | 0.0954 | 0.0188 | 0.3778 | 4.07E-07 | 35.21518 | 0.004279 |
| EOTAXIN | rs112347425 | T | C | 0.1595 | 0.0276 | 0.0978 | 7.77E-09 | 36.95695 | 0.004489 |
| EOTAXIN | rs12075 | A | G | 0.1692 | 0.0155 | 0.5229 | 1.21E-27 | 118.7561 | 0.014284 |
| EOTAXIN | rs147287945 | A | G | -0.1512 | 0.0313 | 0.0741 | 1.36E-06 | 25.78869 | 0.003137 |
| EOTAXIN | rs187131 | C | G | 0.1264 | 0.0253 | 0.8902 | 5.74E-07 | 25.67566 | 0.003123 |
| EOTAXIN | rs2024050 | A | G | 0.164 | 0.0302 | 0.0739 | 5.47E-08 | 30.28104 | 0.003681 |
| EOTAXIN | rs2027855 | T | C | 0.0743 | 0.0162 | 0.6153 | 4.27E-06 | 21.47347 | 0.002613 |
| EOTAXIN | rs2211994 | T | C | 0.0876 | 0.0177 | 0.276 | 6.98E-07 | 25.20976 | 0.003067 |
| EOTAXIN | rs2228467 | T | C | -0.4154 | 0.0291 | 0.9215 | 3.47E-46 | 209.8247 | 0.024965 |
| EOTAXIN | rs5754733 | A | C | -0.105 | 0.0213 | 0.8306 | 8.20E-07 | 25.50424 | 0.003103 |
| EOTAXIN | rs59808887 | T | C | -0.1698 | 0.0356 | 0.0601 | 1.89E-06 | 26.78104 | 0.003257 |
| EOTAXIN | rs745331 | A | G | -0.0821 | 0.0176 | 0.6825 | 3.04E-06 | 24.00945 | 0.002921 |
| EOTAXIN | rs75426604 | A | C | -0.1371 | 0.0291 | 0.0876 | 2.40E-06 | 24.69734 | 0.003005 |
| EOTAXIN | rs79722574 | T | C | -0.1092 | 0.0227 | 0.1422 | 1.50E-06 | 23.90975 | 0.002909 |
| EOTAXIN | rs80341932 | A | G | 0.101 | 0.0204 | 0.8094 | 7.40E-07 | 25.87479 | 0.003147 |
| EOTAXIN | rs9317045 | A | C | 0.1172 | 0.0236 | 0.8615 | 6.95E-07 | 26.95039 | 0.003278 |
| FGF-BASIC | rs116745220 | A | G | -0.6176 | 0.1324 | 0.9951 | 3.09E-06 | 28.40137 | 0.00372 |
| FGF-BASIC | rs13412535 | A | G | -0.1129 | 0.0224 | 0.1856 | 4.76E-07 | 29.42549 | 0.003853 |
| FGF-BASIC | rs61990749 | C | G | 0.1124 | 0.0228 | 0.1545 | 8.23E-07 | 25.1915 | 0.003301 |
| FGF-BASIC | rs75168112 | T | C | -0.1024 | 0.0214 | 0.8097 | 1.64E-06 | 24.66107 | 0.003231 |
| FGF-BASIC | rs78873483 | A | G | 0.1286 | 0.0282 | 0.1025 | 4.98E-06 | 23.21706 | 0.003043 |
| FGF-BASIC | rs9903590 | T | C | 0.1281 | 0.0267 | 0.8953 | 1.62E-06 | 23.47443 | 0.003076 |
| G-CSF | rs115256310 | A | G | -0.6788 | 0.1359 | 0.9954 | 5.85E-07 | 33.67085 | 0.00422 |
| G-CSF | rs117261691 | T | C | 0.1318 | 0.0288 | 0.0894 | 4.67E-06 | 22.53744 | 0.002828 |
| G-CSF | rs11903143 | A | G | 0.0889 | 0.0175 | 0.7066 | 3.78E-07 | 26.1241 | 0.003277 |
| G-CSF | rs145756094 | C | G | -0.7323 | 0.1479 | 0.9962 | 7.40E-07 | 26.09881 | 0.00406 |
| G-CSF | rs2671444 | A | G | -0.0776 | 0.0166 | 0.6506 | 2.86E-06 | 21.81371 | 0.002738 |
| G-CSF | rs586313 | T | C | -0.0883 | 0.0187 | 0.2417 | 2.36E-06 | 22.77512 | 0.002858 |
| G-CSF | rs74148555 | T | C | -0.3771 | 0.0753 | 0.0144 | 5.59E-07 | 32.20412 | 0.004037 |
| G-CSF | rs76287671 | T | C | 0.0894 | 0.0189 | 0.2333 | 2.19E-06 | 22.78439 | 0.002859 |
| G-CSF | rs77318030 | T | C | -0.2031 | 0.0427 | 0.9563 | 2.02E-06 | 27.48995 | 0.003448 |
| GROA | rs1113500 | T | G | 0.1162 | 0.0243 | 0.5981 | 1.72E-06 | 23.14254 | 0.006491 |
| GROA | rs114991247 | T | C | -0.2202 | 0.0463 | 0.9226 | 1.97E-06 | 24.69935 | 0.006925 |
| GROA | rs118158560 | A | G | 0.2761 | 0.0592 | 0.043 | 3.09E-06 | 22.36274 | 0.006274 |
| GROA | rs12075 | A | G | 0.3724 | 0.0236 | 0.5354 | 3.46E-56 | 262.4839 | 0.068993 |
| GROA | rs140734053 | A | G | 0.7333 | 0.1545 | 0.0066 | 2.07E-06 | 25.15262 | 0.007051 |
| GROA | rs185768063 | A | G | 0.4038 | 0.076 | 0.9735 | 1.06E-07 | 30.05122 | 0.008413 |
| GROA | rs188345231 | T | C | 0.6177 | 0.1322 | 0.0102 | 2.97E-06 | 27.50048 | 0.007704 |
| GROA | rs508977 | T | G | -0.3838 | 0.0279 | 0.7566 | 4.57E-43 | 203.1892 | 0.054253 |
| GROA | rs62024303 | A | G | -0.3013 | 0.066 | 0.9617 | 4.91E-06 | 23.84676 | 0.006688 |
| GROA | rs76390238 | C | G | 0.6223 | 0.1352 | 0.0095 | 4.14E-06 | 26.00357 | 0.007288 |
| GROA | rs78653452 | T | G | -0.7395 | 0.1559 | 0.0071 | 2.09E-06 | 27.52202 | 0.00771 |
| HGF | rs11060254 | A | G | -0.0765 | 0.0166 | 0.3306 | 3.97E-06 | 21.64319 | 0.00259 |
| HGF | rs13412535 | A | G | -0.1043 | 0.0213 | 0.1892 | 9.67E-07 | 27.90865 | 0.003338 |
| HGF | rs1617833 | C | G | -0.0749 | 0.016 | 0.6189 | 2.93E-06 | 22.1135 | 0.002646 |
| HGF | rs180840563 | A | T | -0.2022 | 0.0416 | 0.0402 | 1.15E-06 | 26.37697 | 0.003155 |
| HGF | rs2003620 | T | C | 0.2277 | 0.0487 | 0.027 | 2.98E-06 | 22.76517 | 0.002724 |
| HGF | rs3748034 | T | G | 0.1529 | 0.0233 | 0.1278 | 5.21E-11 | 43.66312 | 0.005212 |
| HGF | rs4245058 | T | C | -0.1552 | 0.0331 | 0.0619 | 2.68E-06 | 23.37886 | 0.002797 |
| HGF | rs57146176 | A | G | -0.0987 | 0.0208 | 0.7998 | 2.18E-06 | 26.08076 | 0.00312 |
| HGF | rs5745687 | T | C | -0.3008 | 0.0404 | 0.0375 | 9.92E-14 | 54.79199 | 0.006532 |
| IFN-G | rs10481651 | A | G | -0.0793 | 0.0168 | 0.6116 | 2.18E-06 | 23.20234 | 0.002988 |
| IFN-G | rs113600793 | A | C | 0.1871 | 0.0371 | 0.0631 | 4.43E-07 | 32.18183 | 0.004139 |
| IFN-G | rs115729819 | A | G | 0.2511 | 0.0514 | 0.9716 | 1.05E-06 | 27.03662 | 0.00348 |
| IFN-G | rs11843756 | T | G | 0.1812 | 0.0391 | 0.9542 | 3.62E-06 | 22.2848 | 0.00287 |
| IFN-G | rs12420286 | T | C | 0.2357 | 0.05 | 0.9699 | 2.45E-06 | 25.19782 | 0.003244 |
| IFN-G | rs2073438 | A | G | 0.092 | 0.0188 | 0.2506 | 9.55E-07 | 24.69406 | 0.003179 |
| IFN-G | rs2188420 | C | G | 0.1005 | 0.0201 | 0.7912 | 5.90E-07 | 25.92626 | 0.003337 |
| IFN-G | rs60059008 | A | G | 0.0852 | 0.0176 | 0.6862 | 1.30E-06 | 24.28186 | 0.003126 |
| IFN-G | rs73479333 | C | G | -0.1123 | 0.024 | 0.1354 | 2.82E-06 | 22.93068 | 0.002953 |
| IFN-G | rs74148555 | T | C | -0.3771 | 0.077 | 0.0144 | 9.86E-07 | 31.38138 | 0.004037 |
| IFN-G | rs78296352 | T | G | 0.3419 | 0.065 | 0.0186 | 1.42E-07 | 33.18592 | 0.004268 |
| IL-10 | rs10457128 | A | G | -0.0854 | 0.0172 | 0.639 | 6.96E-07 | 26.07038 | 0.003365 |
| IL-10 | rs10493718 | A | C | -0.1081 | 0.0222 | 0.1603 | 1.07E-06 | 24.36897 | 0.003146 |
| IL-10 | rs10888839 | C | G | 0.1203 | 0.025 | 0.1208 | 1.56E-06 | 23.81128 | 0.003074 |
| IL-10 | rs1530455 | T | C | 0.082 | 0.0174 | 0.3523 | 2.53E-06 | 23.76888 | 0.003069 |
| IL-10 | rs2086656 | T | C | -0.08 | 0.017 | 0.6621 | 2.59E-06 | 22.1767 | 0.002864 |
| IL-10 | rs282258 | T | C | 0.0993 | 0.0162 | 0.4311 | 8.63E-10 | 37.52994 | 0.004837 |
| IL-10 | rs3002131 | C | G | 0.1191 | 0.026 | 0.1195 | 4.59E-06 | 23.11953 | 0.002985 |
| IL-10 | rs3025021 | T | C | 0.0913 | 0.0194 | 0.3459 | 2.61E-06 | 29.2373 | 0.003772 |
| IL-10 | rs339203 | T | C | 0.0954 | 0.0203 | 0.8359 | 2.75E-06 | 14.2826 | 0.002497 |
| IL-10 | rs383684 | A | G | 0.092 | 0.0197 | 0.7555 | 3.17E-06 | 24.22194 | 0.003127 |
| IL-10 | rs41282660 | A | G | -0.1169 | 0.0254 | 0.8646 | 4.23E-06 | 24.78646 | 0.0032 |
| IL-10 | rs6085948 | A | G | 0.0977 | 0.0202 | 0.7946 | 1.28E-06 | 24.13533 | 0.003116 |
| IL-10 | rs6799107 | T | C | -0.095 | 0.0206 | 0.8089 | 3.99E-06 | 21.60608 | 0.00279 |
| IL-10 | rs6921438 | A | G | -0.2876 | 0.0166 | 0.4815 | 1.38E-67 | 332.6596 | 0.0413 |
| IL-10 | rs7088799 | T | G | -0.0815 | 0.0166 | 0.6191 | 9.35E-07 | 24.26663 | 0.003133 |
| IL-12 | rs13209117 | A | G | 0.0981 | 0.0186 | 0.2453 | 1.27E-07 | 29.72321 | 0.003563 |
| IL-12 | rs2123852 | T | C | 0.0942 | 0.0204 | 0.2039 | 3.73E-06 | 24.01459 | 0.002881 |
| IL-12 | rs273702 | A | G | -0.127 | 0.027 | 0.9015 | 2.52E-06 | 23.8776 | 0.002864 |
| IL-12 | rs282258 | T | C | 0.0726 | 0.0156 | 0.4279 | 3.28E-06 | 21.50528 | 0.002581 |
| IL-12 | rs34291323 | T | C | 0.0954 | 0.0198 | 0.6454 | 1.49E-06 | 26.3248 | 0.004166 |
| IL-12 | rs41282644 | A | G | 0.1401 | 0.0303 | 0.0849 | 3.74E-06 | 25.42814 | 0.00305 |
| IL-12 | rs6532374 | T | C | -0.1033 | 0.0226 | 0.8525 | 4.61E-06 | 22.36607 | 0.002684 |
| IL-12 | rs6921438 | A | G | -0.3784 | 0.016 | 0.4834 | 5.78E-124 | 640.2118 | 0.071514 |
| IL-12 | rs6993770 | A | T | 0.0918 | 0.0188 | 0.7786 | 1.06E-06 | 24.22013 | 0.002905 |
| IL-12 | rs71361173 | T | G | 0.1105 | 0.0238 | 0.878 | 3.57E-06 | 21.79977 | 0.002616 |
| IL-12 | rs72831623 | A | G | 0.1929 | 0.0367 | 0.0645 | 1.51E-07 | 37.49368 | 0.004491 |
| IL-12 | rs782107 | A | G | 0.0765 | 0.0156 | 0.4709 | 9.13E-07 | 24.31046 | 0.002916 |
| IL-12 | rs9472183 | A | G | -0.1006 | 0.0157 | 0.4566 | 1.38E-10 | 41.95402 | 0.005022 |
| IL-13 | rs10995615 | T | C | -0.1591 | 0.0341 | 0.8577 | 3.12E-06 | 22.34501 | 0.006179 |
| IL-13 | rs117795020 | A | G | -0.3584 | 0.0716 | 0.0304 | 5.48E-07 | 27.42278 | 0.007572 |
| IL-13 | rs12623722 | A | G | -0.1189 | 0.0257 | 0.3054 | 3.61E-06 | 21.68644 | 0.005998 |
| IL-13 | rs138854806 | A | G | -0.4204 | 0.0839 | 0.0247 | 5.45E-07 | 30.86616 | 0.008515 |
| IL-13 | rs139083458 | T | C | 0.9995 | 0.211 | 0.0034 | 2.17E-06 | 24.49761 | 0.00677 |
| IL-13 | rs27949 | T | C | -0.1144 | 0.025 | 0.667 | 4.83E-06 | 21.0166 | 0.005814 |
| IL-13 | rs6799107 | T | C | -0.1472 | 0.0299 | 0.8071 | 8.66E-07 | 24.41312 | 0.006747 |
| IL-13 | rs6921438 | A | G | -0.4139 | 0.0242 | 0.48 | 1.28E-65 | 336.1004 | 0.08552 |
| IL-13 | rs7073807 | T | C | 0.1618 | 0.0354 | 0.1351 | 4.77E-06 | 22.12338 | 0.006118 |
| IL-13 | rs75383097 | C | G | -0.5369 | 0.116 | 0.0132 | 3.70E-06 | 27.19391 | 0.00751 |
| IL-13 | rs76339001 | A | T | -0.4375 | 0.0886 | 0.9767 | 7.92E-07 | 31.58503 | 0.008712 |
| IL-13 | rs77955971 | A | C | 0.4408 | 0.0868 | 0.0247 | 3.76E-07 | 33.96341 | 0.009362 |
| IL-16 | rs116135478 | A | G | 0.8296 | 0.1637 | 0.9933 | 4.05E-07 | 32.53409 | 0.009161 |
| IL-16 | rs117217798 | T | C | -0.2064 | 0.044 | 0.0889 | 2.77E-06 | 24.45366 | 0.006901 |
| IL-16 | rs117916513 | A | G | -0.4713 | 0.0982 | 0.0173 | 1.61E-06 | 26.77957 | 0.007553 |
| IL-16 | rs1255143 | T | C | 0.1387 | 0.0241 | 0.5695 | 8.53E-09 | 33.51083 | 0.009433 |
| IL-16 | rs144691581 | A | G | 0.4929 | 0.0958 | 0.0197 | 2.67E-07 | 33.33394 | 0.009384 |
| IL-16 | rs1801020 | A | G | 0.1678 | 0.0271 | 0.2655 | 5.63E-10 | 39.07376 | 0.010982 |
| IL-16 | rs4253283 | T | C | 0.1506 | 0.026 | 0.3087 | 7.22E-09 | 34.3975 | 0.00968 |
| IL-16 | rs4778636 | A | G | -0.7286 | 0.063 | 0.0381 | 6.21E-31 | 142.4684 | 0.03891 |
| IL-16 | rs9706053 | T | C | 0.4412 | 0.0928 | 0.0173 | 1.98E-06 | 23.44614 | 0.006619 |
| IL-17 | rs11640734 | C | G | -0.115 | 0.024 | 0.8673 | 1.61E-06 | 23.82297 | 0.003044 |
| IL-17 | rs117556572 | T | C | -0.5256 | 0.1097 | 0.0158 | 1.66E-06 | 30.79967 | 0.008592 |
| IL-17 | rs12735700 | T | G | -0.0943 | 0.0206 | 0.1925 | 4.50E-06 | 21.62892 | 0.002765 |
| IL-17 | rs148562661 | C | G | 0.2161 | 0.0434 | 0.9605 | 6.37E-07 | 27.7448 | 0.003544 |
| IL-17 | rs149738638 | T | C | -0.1553 | 0.0337 | 0.9382 | 4.11E-06 | 21.8816 | 0.002797 |
| IL-17 | rs1530455 | T | C | 0.1088 | 0.0173 | 0.3534 | 3.29E-10 | 42.4377 | 0.00541 |
| IL-17 | rs17106604 | T | C | 0.1119 | 0.0225 | 0.1539 | 6.23E-07 | 25.52554 | 0.003261 |
| IL-17 | rs17282552 | T | C | -0.2026 | 0.0403 | 0.952 | 4.88E-07 | 29.37821 | 0.003751 |
| IL-17 | rs184080173 | T | C | 0.236 | 0.0471 | 0.9693 | 5.39E-07 | 25.94768 | 0.003315 |
| IL-17 | rs78296352 | T | G | 0.2949 | 0.0645 | 0.0186 | 4.81E-06 | 24.84995 | 0.003175 |
| IL-17 | rs9568764 | C | G | 0.0825 | 0.018 | 0.2756 | 4.68E-06 | 21.26098 | 0.002718 |
| IL-18 | rs10414578 | T | C | -0.1817 | 0.0347 | 0.1295 | 1.64E-07 | 27.54508 | 0.007444 |
| IL-18 | rs116383510 | A | C | -0.5412 | 0.1052 | 0.9868 | 2.70E-07 | 28.24204 | 0.00763 |
| IL-18 | rs117266781 | T | C | 0.7051 | 0.1436 | 0.0073 | 9.18E-07 | 26.65839 | 0.007206 |
| IL-18 | rs12420140 | A | G | -0.2479 | 0.0261 | 0.283 | 1.95E-21 | 93.94594 | 0.02494 |
| IL-18 | rs143370787 | C | G | -0.3447 | 0.066 | 0.9554 | 1.75E-07 | 37.5728 | 0.010126 |
| IL-18 | rs17229943 | A | C | -0.3076 | 0.0463 | 0.9176 | 3.06E-11 | 53.31667 | 0.014308 |
| IL-18 | rs1979967 | T | C | 0.14 | 0.0285 | 0.2157 | 8.72E-07 | 24.52048 | 0.006632 |
| IL-18 | rs385076 | T | C | -0.2472 | 0.0247 | 0.3444 | 1.56E-23 | 104.2324 | 0.027595 |
| IL-18 | rs4482818 | A | G | 0.1233 | 0.0243 | 0.609 | 4.11E-07 | 26.78718 | 0.00724 |
| IL-18 | rs610473 | A | G | 0.1274 | 0.0242 | 0.3522 | 1.43E-07 | 27.40618 | 0.007406 |
| IL-18 | rs7444013 | A | G | -0.5318 | 0.0955 | 0.0169 | 2.59E-08 | 34.84437 | 0.009397 |
| IL-18 | rs78623212 | T | C | 0.8322 | 0.1676 | 0.0053 | 6.82E-07 | 27.01825 | 0.007302 |
| IL-18 | rs78716465 | A | G | 0.3173 | 0.0679 | 0.033 | 2.98E-06 | 23.75369 | 0.006426 |
| IL-1B | rs143319329 | T | C | 0.4357 | 0.093 | 0.0209 | 2.84E-06 | 26.19942 | 0.007769 |
| IL-1B | rs61335305 | A | C | 0.4333 | 0.0928 | 0.0189 | 3.02E-06 | 23.4608 | 0.006963 |
| IL-1B | rs62015704 | A | G | 0.1786 | 0.0372 | 0.8688 | 1.62E-06 | 24.50994 | 0.007272 |
| IL-1RA | rs1054402 | T | C | 0.1325 | 0.0269 | 0.2506 | 8.20E-07 | 24.39423 | 0.006594 |
| IL-1RA | rs11627423 | A | C | 0.1178 | 0.0246 | 0.6414 | 1.65E-06 | 23.61013 | 0.006384 |
| IL-1RA | rs11869294 | C | G | -0.2286 | 0.047 | 0.9205 | 1.13E-06 | 28.32469 | 0.007648 |
| IL-1RA | rs3876037 | A | G | 0.1234 | 0.027 | 0.6783 | 4.73E-06 | 24.58591 | 0.006646 |
| IL-1RA | rs4441609 | T | C | 0.1056 | 0.0231 | 0.4757 | 4.75E-06 | 20.55657 | 0.005563 |
| IL-1RA | rs56134659 | A | G | -0.1109 | 0.0236 | 0.4835 | 2.56E-06 | 22.71398 | 0.006143 |
| IL-1RA | rs61335305 | A | C | 0.4315 | 0.0904 | 0.0182 | 1.81E-06 | 24.61744 | 0.006654 |
| IL-1RA | rs6699436 | A | G | -0.1858 | 0.0404 | 0.0995 | 4.37E-06 | 22.87602 | 0.006186 |
| IL-2 | rs13412535 | A | G | 0.174 | 0.0331 | 0.1886 | 1.45E-07 | 32.84753 | 0.009266 |
| IL-2 | rs16836080 | A | G | 0.1158 | 0.0253 | 0.3355 | 4.84E-06 | 21.12485 | 0.005979 |
| IL-2 | rs170117 | T | C | -0.1637 | 0.0347 | 0.1377 | 2.44E-06 | 22.49297 | 0.006364 |
| IL-2 | rs2690020 | A | G | 0.1158 | 0.0245 | 0.5177 | 2.27E-06 | 23.67637 | 0.006696 |
| IL-2 | rs4634519 | A | G | -0.1249 | 0.0268 | 0.7379 | 3.18E-06 | 21.32075 | 0.006034 |
| IL-2 | rs61335305 | A | C | 0.4439 | 0.0913 | 0.0184 | 1.16E-06 | 25.17732 | 0.007118 |
| IL-2 | rs62124990 | T | G | -0.7013 | 0.149 | 0.0083 | 2.50E-06 | 28.66693 | 0.008096 |
| IL-2 | rs7615304 | A | G | -0.1139 | 0.024 | 0.4531 | 2.16E-06 | 22.72664 | 0.00643 |
| IL-2RA | rs11241559 | T | G | -0.124 | 0.0264 | 0.2643 | 2.75E-06 | 22.34179 | 0.00598 |
| IL-2RA | rs117244812 | A | G | -0.7187 | 0.1493 | 0.0079 | 1.47E-06 | 30.31659 | 0.008097 |
| IL-2RA | rs12722497 | A | C | 0.6287 | 0.0482 | 0.0624 | 7.98E-39 | 180.1054 | 0.046251 |
| IL-2RA | rs12799226 | T | C | -0.1285 | 0.0277 | 0.2361 | 3.56E-06 | 22.25385 | 0.005956 |
| IL-2RA | rs28441585 | A | T | 0.1269 | 0.0271 | 0.7688 | 2.93E-06 | 21.38404 | 0.005725 |
| IL-2RA | rs4733117 | A | C | 0.1439 | 0.0291 | 0.7938 | 7.91E-07 | 25.34821 | 0.006779 |
| IL-4 | rs10512267 | T | C | -0.0824 | 0.016 | 0.5752 | 2.73E-07 | 27.18571 | 0.003318 |
| IL-4 | rs116705532 | T | G | -0.4675 | 0.0978 | 0.9925 | 1.73E-06 | 26.65691 | 0.003254 |
| IL-4 | rs12238729 | T | C | 0.5271 | 0.1096 | 0.0162 | 1.51E-06 | 31.78227 | 0.008856 |
| IL-4 | rs13106889 | A | T | -0.1186 | 0.0224 | 0.1648 | 1.22E-07 | 31.74252 | 0.003872 |
| IL-4 | rs17713451 | A | G | 0.1255 | 0.0252 | 0.1149 | 6.41E-07 | 26.24416 | 0.003204 |
| IL-4 | rs2073438 | A | G | 0.0847 | 0.0183 | 0.2513 | 3.73E-06 | 22.10449 | 0.0027 |
| IL-4 | rs6765768 | A | G | 0.0796 | 0.0167 | 0.654 | 1.85E-06 | 23.4837 | 0.002868 |
| IL-4 | rs6969391 | T | C | 0.0767 | 0.0166 | 0.6589 | 3.59E-06 | 21.65117 | 0.002644 |
| IL-4 | rs73023729 | A | G | -0.1796 | 0.0365 | 0.0519 | 8.56E-07 | 26.00485 | 0.003174 |
| IL-4 | rs7613691 | A | G | 0.1787 | 0.0382 | 0.9482 | 2.96E-06 | 25.69702 | 0.003137 |
| IL-4 | rs79597994 | T | C | -0.5855 | 0.1271 | 0.0049 | 4.06E-06 | 27.39115 | 0.003343 |
| IL-4 | rs9508291 | T | C | -0.168 | 0.0358 | 0.9495 | 2.67E-06 | 22.16263 | 0.002707 |
| IL-4 | rs9941733 | A | G | 0.1156 | 0.0229 | 0.8355 | 4.33E-07 | 30.10687 | 0.003673 |
| IL-5 | rs11680908 | A | G | 0.2593 | 0.0552 | 0.9485 | 2.62E-06 | 22.48787 | 0.006569 |
| IL-5 | rs148634917 | A | G | -0.517 | 0.1087 | 0.986 | 1.97E-06 | 25.28363 | 0.007379 |
| IL-5 | rs28793375 | T | C | 0.1697 | 0.0362 | 0.1298 | 2.75E-06 | 22.27043 | 0.006506 |
| IL-5 | rs6737109 | T | C | 0.1135 | 0.0246 | 0.519 | 3.81E-06 | 22.01624 | 0.006432 |
| IL-5 | rs73040130 | T | C | 0.2745 | 0.0525 | 0.9388 | 1.71E-07 | 29.70452 | 0.008658 |
| IL-5 | rs74811276 | A | G | 0.217 | 0.0471 | 0.0761 | 4.08E-06 | 22.66997 | 0.006622 |
| IL-5 | rs9472168 | A | G | 0.1568 | 0.0253 | 0.5389 | 5.42E-10 | 42.06988 | 0.012219 |
| IL-6 | rs10752777 | A | T | 0.1083 | 0.0235 | 0.1273 | 4.17E-06 | 21.50632 | 0.002606 |
| IL-6 | rs10982213 | A | G | -0.0849 | 0.0176 | 0.2723 | 1.35E-06 | 23.57981 | 0.002857 |
| IL-6 | rs113098456 | A | G | -0.1553 | 0.0339 | 0.0725 | 4.64E-06 | 26.7848 | 0.003244 |
| IL-6 | rs1333040 | T | C | 0.0747 | 0.0157 | 0.4508 | 1.99E-06 | 22.80551 | 0.002763 |
| IL-6 | rs13412535 | A | G | -0.1186 | 0.0214 | 0.189 | 3.14E-08 | 35.64605 | 0.004312 |
| IL-6 | rs2404476 | A | G | 0.0734 | 0.0156 | 0.5184 | 2.68E-06 | 22.2022 | 0.00269 |
| IL-6 | rs4684700 | T | C | -0.0747 | 0.0162 | 0.5142 | 3.91E-06 | 23.01049 | 0.002788 |
| IL-6 | rs72831623 | A | G | 0.197 | 0.0369 | 0.0645 | 9.29E-08 | 38.73088 | 0.004683 |
| IL-6 | rs73273528 | T | C | 0.268 | 0.0553 | 0.0211 | 1.25E-06 | 24.49421 | 0.002967 |
| IL-6 | rs75101555 | C | G | -0.3625 | 0.0781 | 0.9895 | 3.44E-06 | 22.53675 | 0.002731 |
| IL-6 | rs76856708 | T | C | 0.336 | 0.0697 | 0.9856 | 1.43E-06 | 26.46173 | 0.003205 |
| IL-7 | rs10196226 | A | G | 0.1538 | 0.0327 | 0.1643 | 2.50E-06 | 22.53078 | 0.006496 |
| IL-7 | rs115215018 | T | C | 0.5985 | 0.1308 | 0.0101 | 4.76E-06 | 24.8604 | 0.007163 |
| IL-7 | rs117509142 | T | C | -0.3213 | 0.0684 | 0.9569 | 2.60E-06 | 29.59542 | 0.008515 |
| IL-7 | rs141425475 | T | C | -0.4801 | 0.1018 | 0.9814 | 2.39E-06 | 29.24406 | 0.008415 |
| IL-7 | rs17091524 | T | C | 0.5092 | 0.1015 | 0.9849 | 5.24E-07 | 26.78265 | 0.007712 |
| IL-7 | rs1958987 | T | C | 0.1261 | 0.0263 | 0.7051 | 1.60E-06 | 22.93942 | 0.006613 |
| IL-7 | rs218247 | C | G | -0.1343 | 0.0285 | 0.2258 | 2.42E-06 | 21.86864 | 0.006306 |
| IL-7 | rs28793375 | T | C | 0.1644 | 0.036 | 0.1305 | 4.87E-06 | 21.26674 | 0.006134 |
| IL-7 | rs62006410 | T | C | -0.1492 | 0.0302 | 0.245 | 7.59E-07 | 28.61458 | 0.008235 |
| IL-7 | rs6921438 | A | G | -0.3204 | 0.0246 | 0.4814 | 8.71E-39 | 186.1746 | 0.051257 |
| IL-7 | rs77981494 | T | C | -0.5201 | 0.1055 | 0.984 | 8.23E-07 | 29.60391 | 0.008518 |
| IL-8 | rs116726256 | T | C | -0.2247 | 0.0489 | 0.9343 | 4.26E-06 | 22.22307 | 0.006199 |
| IL-8 | rs12075 | A | G | 0.1148 | 0.0235 | 0.5353 | 9.97E-07 | 23.51562 | 0.006557 |
| IL-8 | rs12438669 | A | C | -0.1182 | 0.0252 | 0.6834 | 2.60E-06 | 21.67206 | 0.006046 |
| IL-8 | rs141926526 | A | C | -0.6221 | 0.1308 | 0.9916 | 1.96E-06 | 23.12017 | 0.006447 |
| IL-8 | rs183628733 | T | C | 0.6547 | 0.1417 | 0.9917 | 3.82E-06 | 25.32003 | 0.007056 |
| IL-8 | rs2673604 | A | C | -0.118 | 0.0254 | 0.6829 | 3.29E-06 | 21.61673 | 0.00603 |
| IL-8 | rs3786107 | A | G | 0.2463 | 0.0517 | 0.9287 | 1.94E-06 | 28.85644 | 0.008034 |
| IL-8 | rs75840288 | A | C | 0.5125 | 0.1121 | 0.9839 | 4.85E-06 | 29.89781 | 0.008321 |
| IL-9 | rs117807175 | C | G | -0.5225 | 0.1106 | 0.0121 | 2.33E-06 | 24.11733 | 0.006527 |
| IL-9 | rs3736858 | C | G | -0.1351 | 0.0291 | 0.7976 | 3.37E-06 | 21.76144 | 0.005893 |
| IL-9 | rs41294750 | T | C | 0.3442 | 0.0736 | 0.0308 | 2.92E-06 | 26.15068 | 0.007073 |
| IL-9 | rs4880409 | T | C | -0.3552 | 0.0716 | 0.9664 | 6.95E-07 | 30.32701 | 0.008194 |
| IL-9 | rs73443903 | A | C | 0.2162 | 0.046 | 0.0685 | 2.57E-06 | 22.02914 | 0.005965 |
| IL-9 | rs76963786 | T | C | -0.2856 | 0.0556 | 0.0462 | 2.78E-07 | 26.58051 | 0.007189 |
| IP-10 | rs113831257 | A | G | 0.3639 | 0.0641 | 0.0417 | 1.39E-08 | 39.81337 | 0.010584 |
| IP-10 | rs34383175 | T | C | -0.3196 | 0.0653 | 0.035 | 9.90E-07 | 25.85962 | 0.0069 |
| IP-10 | rs397816 | T | C | 0.1211 | 0.0248 | 0.5891 | 1.03E-06 | 26.61425 | 0.0071 |
| IP-10 | rs4862111 | T | C | 0.1448 | 0.0317 | 0.2313 | 4.84E-06 | 27.95929 | 0.007456 |
| IP-10 | rs6707974 | A | G | 0.1574 | 0.0337 | 0.8626 | 3.03E-06 | 21.9872 | 0.005873 |
| IP-10 | rs7645625 | T | G | -0.1116 | 0.0236 | 0.5724 | 2.19E-06 | 22.83116 | 0.006097 |
| IP-10 | rs79848609 | A | C | 0.2514 | 0.0535 | 0.9449 | 2.64E-06 | 24.6571 | 0.006581 |
| IP-10 | rs8112909 | A | G | -0.139 | 0.0297 | 0.8159 | 2.96E-06 | 21.72975 | 0.005804 |
| IP-10 | rs9450351 | T | C | -0.2651 | 0.0488 | 0.9387 | 5.48E-08 | 30.34869 | 0.008088 |
| M-CSF | rs116274860 | T | G | 0.8262 | 0.1739 | 0.9908 | 2.03E-06 | 31.45265 | 0.012444 |
| M-CSF | rs11963606 | C | G | -0.5353 | 0.117 | 0.9826 | 4.73E-06 | 24.69855 | 0.009798 |
| M-CSF | rs12962919 | T | C | 0.3025 | 0.0659 | 0.0644 | 4.39E-06 | 27.83024 | 0.011027 |
| M-CSF | rs34089869 | T | C | 0.2194 | 0.0462 | 0.1039 | 2.08E-06 | 22.57513 | 0.008963 |
| M-CSF | rs4269021 | C | G | -0.2459 | 0.0504 | 0.9101 | 1.05E-06 | 24.94359 | 0.009895 |
| M-CSF | rs56367447 | T | C | -0.4878 | 0.0876 | 0.0305 | 2.57E-08 | 35.62548 | 0.014072 |
| M-CSF | rs62294910 | A | G | 0.3472 | 0.0687 | 0.0504 | 4.38E-07 | 29.13705 | 0.011539 |
| M-CSF | rs72723242 | T | G | -0.4969 | 0.1083 | 0.0177 | 4.43E-06 | 21.61598 | 0.008586 |
| M-CSF | rs78296352 | T | G | 0.522 | 0.111 | 0.0195 | 2.58E-06 | 26.28129 | 0.01042 |
| M-CSF | rs9387100 | T | C | -0.135 | 0.029 | 0.4291 | 3.34E-06 | 22.48827 | 0.008929 |
| MCP-1 | rs10744620 | T | C | 0.0783 | 0.0161 | 0.3748 | 1.12E-06 | 24.01747 | 0.002873 |
| MCP-1 | rs111995966 | T | G | 0.1428 | 0.0309 | 0.9297 | 3.79E-06 | 22.27662 | 0.002666 |
| MCP-1 | rs12073356 | A | G | -0.1436 | 0.031 | 0.0686 | 3.49E-06 | 22.0217 | 0.002635 |
| MCP-1 | rs12075 | A | G | 0.2186 | 0.0154 | 0.523 | 1.36E-45 | 203.5804 | 0.023842 |
| MCP-1 | rs143815843 | A | G | -0.2049 | 0.0447 | 0.0315 | 4.61E-06 | 21.4064 | 0.002562 |
| MCP-1 | rs146522229 | T | C | -0.5942 | 0.1161 | 0.0047 | 3.09E-07 | 27.6242 | 0.003303 |
| MCP-1 | rs2036297 | A | G | 0.1182 | 0.016 | 0.384 | 1.30E-13 | 55.45779 | 0.00661 |
| MCP-1 | rs2288370 | T | C | -0.1036 | 0.0162 | 0.6304 | 1.56E-10 | 41.8968 | 0.005001 |
| MCP-1 | rs56212190 | T | C | 0.1799 | 0.0372 | 0.0519 | 1.32E-06 | 26.63207 | 0.003185 |
| MCP-1 | rs7197349 | A | G | 0.0971 | 0.0206 | 0.8124 | 2.40E-06 | 24.02297 | 0.002874 |
| MCP-1 | rs7517040 | A | G | -0.097 | 0.019 | 0.2334 | 3.41E-07 | 28.15876 | 0.003367 |
| MCP-1 | rs7632755 | A | G | 0.2984 | 0.0315 | 0.0647 | 2.79E-21 | 90.80172 | 0.010777 |
| MCP-1 | rs9317045 | A | C | 0.1157 | 0.0235 | 0.8618 | 8.43E-07 | 26.66269 | 0.003189 |
| MCP-3 | rs10892381 | T | C | 0.2432 | 0.0473 | 0.6629 | 2.69E-07 | 29.78552 | 0.026434 |
| MCP-3 | rs2838065 | A | G | -0.221 | 0.0479 | 0.2719 | 3.92E-06 | 21.63227 | 0.019338 |
| MCP-3 | rs28394764 | A | T | 0.597 | 0.1282 | 0.9696 | 3.19E-06 | 23.54364 | 0.021011 |
| MCP-3 | rs3129806 | T | C | -0.1975 | 0.0433 | 0.5729 | 4.98E-06 | 21.34762 | 0.019089 |
| MCP-3 | rs62492260 | T | G | -0.2802 | 0.0578 | 0.1886 | 1.23E-06 | 27.00927 | 0.024029 |
| MIF | rs113218956 | A | G | -0.8789 | 0.1876 | 0.0044 | 2.82E-06 | 24.05988 | 0.006768 |
| MIF | rs11551183 | C | G | 0.3666 | 0.0795 | 0.9756 | 4.00E-06 | 22.73851 | 0.006398 |
| MIF | rs12594190 | A | G | 0.1321 | 0.0266 | 0.7008 | 6.85E-07 | 26.03028 | 0.007318 |
| MIF | rs13142904 | T | C | -0.2232 | 0.0425 | 0.0896 | 1.47E-07 | 28.93347 | 0.008128 |
| MIF | rs141009259 | T | C | -0.6194 | 0.1285 | 0.9886 | 1.44E-06 | 30.80119 | 0.008648 |
| MIF | rs2294689 | C | G | -0.1338 | 0.0287 | 0.3018 | 3.04E-06 | 26.84282 | 0.007545 |
| MIF | rs2330634 | C | G | 0.1549 | 0.0249 | 0.3681 | 4.57E-10 | 39.85838 | 0.011162 |
| MIF | rs35890933 | T | G | 0.1676 | 0.0365 | 0.8474 | 4.46E-06 | 25.83957 | 0.007265 |
| MIF | rs3814097 | A | G | -0.1163 | 0.0251 | 0.5533 | 3.55E-06 | 23.76716 | 0.006686 |
| MIF | rs78098071 | T | C | -0.4583 | 0.0915 | 0.9809 | 5.51E-07 | 28.01025 | 0.00787 |
| MIG | rs111607343 | A | G | -0.5235 | 0.1119 | 0.0134 | 2.93E-06 | 27.16715 | 0.007246 |
| MIG | rs11177248 | A | G | 0.3157 | 0.0667 | 0.0351 | 2.22E-06 | 25.29804 | 0.006751 |
| MIG | rs112861654 | A | G | -0.2682 | 0.0527 | 0.9454 | 3.64E-07 | 27.84641 | 0.007426 |
| MIG | rs117831247 | T | C | -0.8819 | 0.173 | 0.0056 | 3.45E-07 | 32.52164 | 0.008662 |
| MIG | rs13143163 | C | G | 0.2735 | 0.0582 | 0.0512 | 2.62E-06 | 27.24793 | 0.007268 |
| MIG | rs139010077 | T | C | 0.4337 | 0.0943 | 0.0169 | 4.19E-06 | 23.40952 | 0.00625 |
| MIG | rs1796086 | T | C | -0.2172 | 0.04 | 0.9127 | 5.62E-08 | 28.19328 | 0.007518 |
| MIG | rs191555775 | A | T | 0.2279 | 0.0412 | 0.909 | 3.28E-08 | 32.25879 | 0.008593 |
| MIG | rs55876513 | T | G | 0.1638 | 0.0254 | 0.7131 | 1.05E-10 | 41.31515 | 0.010978 |
| MIG | rs62562991 | A | G | 0.6239 | 0.1259 | 0.0097 | 7.24E-07 | 28.04367 | 0.007478 |
| MIG | rs6679677 | A | C | 0.1628 | 0.0327 | 0.1458 | 6.51E-07 | 24.73483 | 0.006602 |
| MIG | rs77086208 | T | C | 0.327 | 0.0694 | 0.0286 | 2.50E-06 | 22.24611 | 0.005941 |
| MIG | rs816960 | T | C | -0.1179 | 0.0242 | 0.3692 | 1.15E-06 | 24.2554 | 0.006475 |
| MIP-1A | rs12690897 | A | G | 0.1215 | 0.026 | 0.292 | 3.07E-06 | 21.85675 | 0.006104 |
| MIP-1A | rs184154340 | A | G | 0.3251 | 0.0689 | 0.0304 | 2.40E-06 | 22.31375 | 0.006231 |
| MIP-1A | rs57786342 | A | G | 0.139 | 0.0283 | 0.2336 | 8.91E-07 | 24.7931 | 0.006918 |
| MIP-1A | rs60198979 | A | G | -0.2154 | 0.0455 | 0.0788 | 2.22E-06 | 24.13597 | 0.006736 |
| MIP-1A | rs6900267 | A | C | -0.2472 | 0.0515 | 0.9308 | 1.60E-06 | 28.23903 | 0.007872 |
| MIP-1B | rs113010081 | T | C | -0.5799 | 0.0236 | 0.867 | 1.57E-133 | 696.5603 | 0.077554 |
| MIP-1B | rs113877493 | T | C | -0.607 | 0.0217 | 0.1645 | 3.67E-172 | 933.6571 | 0.101279 |
| MIP-1B | rs117453826 | A | G | -0.5907 | 0.0591 | 0.981 | 1.53E-23 | 109.1855 | 0.013007 |
| MIP-1B | rs117657747 | A | G | 0.2089 | 0.0453 | 0.0526 | 4.01E-06 | 27.37218 | 0.004349 |
| MIP-1B | rs141102180 | T | G | 0.3298 | 0.0392 | 0.0449 | 3.75E-17 | 78.01704 | 0.009329 |
| MIP-1B | rs1437220 | T | C | 0.1437 | 0.0315 | 0.9207 | 4.92E-06 | 25.05757 | 0.003015 |
| MIP-1B | rs17138331 | A | G | -0.1434 | 0.0295 | 0.9092 | 1.13E-06 | 28.2256 | 0.003395 |
| MIP-1B | rs2411161 | T | C | 0.1719 | 0.0365 | 0.9508 | 2.55E-06 | 22.96841 | 0.002765 |
| MIP-1B | rs281748 | C | G | -0.0794 | 0.0171 | 0.7041 | 3.28E-06 | 21.82153 | 0.002627 |
| MIP-1B | rs3760440 | A | G | 0.1242 | 0.0162 | 0.6437 | 1.73E-14 | 59.04035 | 0.007076 |
| MIP-1B | rs5743614 | T | C | 0.1115 | 0.0232 | 0.1278 | 1.57E-06 | 23.02632 | 0.002772 |
| MIP-1B | rs6908843 | A | G | 0.0997 | 0.0209 | 0.1684 | 1.78E-06 | 23.13024 | 0.002784 |
| MIP-1B | rs72791296 | T | C | 0.2364 | 0.0466 | 0.0349 | 3.97E-07 | 31.30785 | 0.003765 |
| MIP-1B | rs72799710 | T | C | -0.1037 | 0.0217 | 0.1514 | 1.79E-06 | 22.95676 | 0.002763 |
| MIP-1B | rs76356863 | A | T | -0.3456 | 0.0667 | 0.9852 | 2.22E-07 | 28.95819 | 0.003483 |
| MIP-1B | rs76582507 | A | G | 0.3259 | 0.0676 | 0.0156 | 1.42E-06 | 27.1148 | 0.003262 |
| MIP-1B | rs76776296 | A | G | 0.313 | 0.0598 | 0.9806 | 1.63E-07 | 30.9975 | 0.003727 |
| MIP-1B | rs79068918 | C | G | 0.2674 | 0.0271 | 0.0909 | 5.54E-23 | 99.07948 | 0.011818 |
| PDGF-BB | rs11247305 | C | G | -0.1687 | 0.0364 | 0.9376 | 3.47E-06 | 27.84945 | 0.00333 |
| PDGF-BB | rs116445074 | T | G | 0.2869 | 0.0587 | 0.0184 | 1.02E-06 | 24.85663 | 0.002973 |
| PDGF-BB | rs11766649 | A | G | 0.0902 | 0.0196 | 0.8018 | 3.96E-06 | 21.60941 | 0.002586 |
| PDGF-BB | rs12289510 | A | G | -0.0772 | 0.0158 | 0.4767 | 1.00E-06 | 24.85761 | 0.002973 |
| PDGF-BB | rs13037046 | A | T | -0.0948 | 0.0206 | 0.1871 | 3.96E-06 | 22.8482 | 0.002734 |
| PDGF-BB | rs13412535 | A | G | 0.3317 | 0.0214 | 0.1892 | 2.89E-54 | 291.1889 | 0.033756 |
| PDGF-BB | rs2324229 | T | C | 0.0884 | 0.0161 | 0.6134 | 4.02E-08 | 31.0069 | 0.003706 |
| PDGF-BB | rs35859699 | A | G | -0.3854 | 0.0838 | 0.0109 | 4.22E-06 | 26.78051 | 0.003203 |
| PDGF-BB | rs4965869 | T | C | 0.1843 | 0.0181 | 0.2446 | 2.22E-24 | 105.9511 | 0.012552 |
| PDGF-BB | rs55680718 | T | C | -0.1359 | 0.0245 | 0.1173 | 2.96E-08 | 31.99999 | 0.003825 |
| PDGF-BB | rs72777070 | T | G | -0.1048 | 0.02 | 0.7854 | 1.56E-07 | 30.97346 | 0.003702 |
| PDGF-BB | rs73162807 | A | C | -0.2313 | 0.0499 | 0.0259 | 3.55E-06 | 22.5613 | 0.0027 |
| PDGF-BB | rs9936075 | A | G | -0.0767 | 0.0163 | 0.6436 | 2.68E-06 | 22.55557 | 0.002699 |
| PDGF-BB | rs9941733 | A | G | 0.1165 | 0.0227 | 0.8364 | 3.02E-07 | 31.07427 | 0.003714 |
| RANTES | rs112072646 | A | G | 0.4209 | 0.0859 | 0.0204 | 9.62E-07 | 24.61634 | 0.007081 |
| RANTES | rs147509526 | T | C | -0.3558 | 0.0715 | 0.0333 | 6.57E-07 | 28.36631 | 0.00815 |
| RANTES | rs2251660 | A | C | 0.1831 | 0.0356 | 0.8627 | 2.69E-07 | 27.63572 | 0.007942 |
| RANTES | rs2731672 | T | C | -0.1242 | 0.0272 | 0.267 | 4.83E-06 | 20.96956 | 0.006038 |
| RANTES | rs62438851 | A | G | -0.1904 | 0.0413 | 0.8961 | 4.01E-06 | 23.4611 | 0.00675 |
| RANTES | rs7000423 | T | C | -0.1314 | 0.0252 | 0.6274 | 1.85E-07 | 28.09305 | 0.008073 |
| RANTES | rs7170339 | C | G | -0.4283 | 0.0904 | 0.0197 | 2.19E-06 | 24.6326 | 0.007085 |
| RANTES | rs72793342 | A | G | -0.1505 | 0.0307 | 0.2003 | 9.08E-07 | 25.23159 | 0.007256 |
| RANTES | rs74472919 | T | C | 0.3547 | 0.06 | 0.0434 | 3.35E-09 | 36.44215 | 0.010447 |
| RANTES | rs9675798 | T | G | -0.2583 | 0.0552 | 0.9466 | 2.89E-06 | 23.44211 | 0.006745 |
| SCF | rs113127926 | A | C | 0.1974 | 0.0418 | 0.0418 | 2.34E-06 | 26.08938 | 0.003121 |
| SCF | rs13412535 | A | G | -0.1065 | 0.0213 | 0.1892 | 5.59E-07 | 29.0956 | 0.00348 |
| SCF | rs1557570 | T | G | 0.1172 | 0.0169 | 0.2954 | 4.13E-12 | 47.91572 | 0.005718 |
| SCF | rs1568119 | T | C | -0.5946 | 0.1129 | 0.0051 | 1.37E-07 | 30.00127 | 0.003588 |
| SCF | rs4841899 | T | C | -0.1002 | 0.0178 | 0.7421 | 1.67E-08 | 32.14406 | 0.003843 |
| SCF | rs635634 | T | C | -0.1035 | 0.0191 | 0.206 | 5.71E-08 | 29.30031 | 0.003504 |
| SCF | rs7039247 | C | G | 0.079 | 0.0168 | 0.6644 | 2.46E-06 | 23.25388 | 0.002783 |
| SCF | rs72678285 | A | T | 0.1062 | 0.0231 | 0.8512 | 4.43E-06 | 23.87291 | 0.002857 |
| SCF | rs78369473 | T | C | -0.2256 | 0.0484 | 0.0266 | 3.14E-06 | 22.01793 | 0.002636 |
| SCF | rs78666213 | T | G | -0.2845 | 0.0574 | 0.9797 | 7.15E-07 | 26.91122 | 0.003219 |
| SCF | rs80271436 | A | G | -0.2393 | 0.0484 | 0.0263 | 7.49E-07 | 24.50875 | 0.002933 |
| SCGF-B | rs112346514 | T | C | -0.3261 | 0.0703 | 0.0302 | 3.54E-06 | 23.31098 | 0.006229 |
| SCGF-B | rs1149926 | T | C | -0.3458 | 0.0749 | 0.0239 | 3.92E-06 | 20.86547 | 0.005579 |
| SCGF-B | rs116924815 | T | C | 0.6046 | 0.0737 | 0.0273 | 2.25E-16 | 73.62888 | 0.019414 |
| SCGF-B | rs117716477 | A | C | 0.8242 | 0.084 | 0.0199 | 1.03E-22 | 101.2297 | 0.026498 |
| SCGF-B | rs12480722 | T | C | 0.1654 | 0.0353 | 0.8752 | 2.81E-06 | 22.359 | 0.005976 |
| SCGF-B | rs13287050 | A | T | -0.121 | 0.0263 | 0.7202 | 4.12E-06 | 22.07486 | 0.005901 |
| SCGF-B | rs139413256 | A | G | -0.5174 | 0.1076 | 0.0139 | 1.53E-06 | 27.49436 | 0.007339 |
| SCGF-B | rs143829871 | T | C | -0.1866 | 0.0399 | 0.9047 | 2.85E-06 | 22.46427 | 0.006004 |
| SCGF-B | rs149009264 | A | G | 0.4551 | 0.0985 | 0.9848 | 3.79E-06 | 23.20399 | 0.006201 |
| SCGF-B | rs150733161 | T | C | -0.5255 | 0.112 | 0.0142 | 2.69E-06 | 28.97674 | 0.007731 |
| SCGF-B | rs151194174 | A | G | 0.4536 | 0.0941 | 0.0187 | 1.45E-06 | 28.29682 | 0.007551 |
| SCGF-B | rs17876031 | A | G | -0.1496 | 0.0254 | 0.324 | 3.67E-09 | 36.8205 | 0.009804 |
| SCGF-B | rs264157 | A | G | 0.1079 | 0.0233 | 0.4743 | 3.69E-06 | 21.71796 | 0.005806 |
| SCGF-B | rs34911860 | A | G | -0.3674 | 0.0787 | 0.0291 | 3.00E-06 | 28.58428 | 0.007627 |
| SCGF-B | rs4656185 | A | G | 0.2103 | 0.0254 | 0.2892 | 1.29E-16 | 68.87311 | 0.018183 |
| SCGF-B | rs77954165 | T | C | 0.2631 | 0.0562 | 0.9528 | 2.87E-06 | 23.2999 | 0.006226 |
| SCGF-B | rs7815967 | T | C | 0.1325 | 0.0288 | 0.1991 | 4.37E-06 | 20.93996 | 0.005599 |
| SCGF-B | rs78217154 | T | C | 0.3942 | 0.0861 | 0.9796 | 4.72E-06 | 23.24203 | 0.006211 |
| SDF-1A | rs10013755 | A | T | 0.5188 | 0.0995 | 0.9929 | 1.85E-07 | 29.81538 | 0.003795 |
| SDF-1A | rs10474392 | A | G | 0.0934 | 0.0177 | 0.334 | 1.38E-07 | 30.49499 | 0.003881 |
| SDF-1A | rs149893336 | A | G | -0.494 | 0.1082 | 0.993 | 4.93E-06 | 26.64418 | 0.003393 |
| SDF-1A | rs1600396 | A | G | -0.0933 | 0.0204 | 0.8096 | 4.94E-06 | 21.06169 | 0.002684 |
| SDF-1A | rs3988298 | T | C | -0.1263 | 0.0266 | 0.1042 | 2.12E-06 | 23.37793 | 0.002978 |
| SDF-1A | rs62194947 | T | C | -0.0852 | 0.0185 | 0.2632 | 4.27E-06 | 22.09859 | 0.002815 |
| SDF-1A | rs78883416 | C | G | -0.0871 | 0.0182 | 0.308 | 1.76E-06 | 25.39365 | 0.003234 |
| TNF-A | rs10834997 | A | G | -0.123 | 0.0256 | 0.6924 | 1.53E-06 | 22.64338 | 0.006444 |
| TNF-A | rs111332265 | A | G | -0.3678 | 0.0745 | 0.9696 | 7.91E-07 | 28.06382 | 0.007975 |
| TNF-A | rs79105320 | A | G | 0.5573 | 0.1177 | 0.0112 | 2.21E-06 | 24.18145 | 0.006879 |
| TNF-B | rs10925040 | T | C | 0.1738 | 0.0372 | 0.371 | 2.93E-06 | 22.36439 | 0.014098 |
| TNF-B | rs75240021 | C | G | 0.3713 | 0.0772 | 0.071 | 1.49E-06 | 28.97089 | 0.018187 |
| TNF-B | rs753274 | T | C | -0.1725 | 0.037 | 0.6085 | 3.14E-06 | 22.49254 | 0.014178 |
| TNF-B | rs7629875 | A | G | 0.3841 | 0.0774 | 0.9443 | 6.90E-07 | 24.65548 | 0.01552 |
| TNF-B | rs78296352 | T | G | 1.2028 | 0.1366 | 0.0252 | 1.28E-18 | 119.6714 | 0.071078 |
| TRAIL | rs13278062 | T | G | 0.08 | 0.0157 | 0.5589 | 3.33E-07 | 26.04642 | 0.003156 |
| TRAIL | rs148051545 | T | C | -0.4211 | 0.0843 | 0.0114 | 5.84E-07 | 33.01867 | 0.003997 |
| TRAIL | rs17434886 | T | C | -0.0918 | 0.0199 | 0.1949 | 4.20E-06 | 21.81833 | 0.002645 |
| TRAIL | rs193112415 | T | C | -1.0456 | 0.062 | 0.9809 | 1.01E-63 | 351.4628 | 0.040966 |
| TRAIL | rs28431810 | C | G | -0.1216 | 0.0252 | 0.8874 | 1.41E-06 | 24.38566 | 0.002955 |
| TRAIL | rs28521641 | A | T | -0.7004 | 0.0445 | 0.963 | 7.79E-56 | 298.0564 | 0.034958 |
| TRAIL | rs550057 | T | C | -0.0783 | 0.0169 | 0.3049 | 3.71E-06 | 21.43792 | 0.002599 |
| TRAIL | rs57396456 | T | C | -0.5641 | 0.0516 | 0.9755 | 7.71E-28 | 127.0827 | 0.01521 |
| TRAIL | rs62093514 | T | C | 1.0459 | 0.0549 | 0.0239 | 5.80E-81 | 442.5359 | 0.051039 |
| TRAIL | rs72899452 | T | C | 0.1223 | 0.0264 | 0.0977 | 3.75E-06 | 21.75552 | 0.002637 |
| TRAIL | rs73039026 | A | C | -0.3098 | 0.0634 | 0.9821 | 1.02E-06 | 27.85889 | 0.003374 |
| TRAIL | rs747324 | T | C | -0.0826 | 0.0178 | 0.2828 | 3.34E-06 | 22.83535 | 0.002768 |
| TRAIL | rs74778900 | T | C | 0.5791 | 0.0531 | 0.0238 | 9.90E-28 | 130.2471 | 0.015583 |
| TRAIL | rs75928541 | A | G | 0.2784 | 0.0591 | 0.0188 | 2.44E-06 | 23.5951 | 0.002859 |
| TRAIL | rs79287178 | A | G | -0.4304 | 0.042 | 0.0389 | 1.17E-24 | 115.5699 | 0.013851 |
| VEGF | rs10411345 | C | G | -0.1041 | 0.0218 | 0.7823 | 1.73E-06 | 26.52664 | 0.003691 |
| VEGF | rs10934631 | T | C | -0.1132 | 0.0244 | 0.8517 | 3.61E-06 | 23.25261 | 0.003237 |
| VEGF | rs10967186 | T | C | 0.0899 | 0.0169 | 0.578 | 1.09E-07 | 28.34121 | 0.003943 |
| VEGF | rs12456390 | T | C | -0.0818 | 0.0179 | 0.6772 | 4.88E-06 | 21.00741 | 0.002925 |
| VEGF | rs13209117 | A | G | 0.1253 | 0.02 | 0.2474 | 3.70E-10 | 42.10713 | 0.005847 |
| VEGF | rs143479231 | A | G | -0.2628 | 0.0489 | 0.0363 | 7.90E-08 | 34.76529 | 0.004832 |
| VEGF | rs3108686 | A | C | -0.7967 | 0.1702 | 0.0038 | 2.86E-06 | 24.82501 | 0.004806 |
| VEGF | rs4082730 | A | G | 0.2455 | 0.0533 | 0.0305 | 4.12E-06 | 25.61205 | 0.003564 |
| VEGF | rs6921438 | A | G | -0.4866 | 0.0174 | 0.4691 | 4.11E-172 | 957.3397 | 0.117938 |
| VEGF | rs7030781 | A | T | 0.1403 | 0.0172 | 0.5878 | 3.45E-16 | 68.95382 | 0.009539 |
| VEGF | rs73418461 | A | G | -0.2498 | 0.0521 | 0.0266 | 1.61E-06 | 23.21168 | 0.003231 |
| VEGF | rs73872715 | T | C | -0.6079 | 0.1299 | 0.0045 | 2.86E-06 | 23.7849 | 0.003311 |
| VEGF | rs8045833 | A | G | 0.103 | 0.0211 | 0.2132 | 1.01E-06 | 25.57511 | 0.003559 |
| VEGF | rs9472183 | A | G | -0.1264 | 0.017 | 0.4547 | 9.54E-14 | 57.18106 | 0.007923 |

## Supplementary Table 6 Detailed information for risk of cisplatin-induced hearing loss in children associated SNPs with circulating cytokines

| Outcome | SNP | Alt | Ref | Beta | SE | EAF | p-val | F | R^2^ |
| --- | --- | --- | --- | --- | --- | --- | --- | --- | --- |
| B-NGF | rs10928931 | C | G | 1.641 | 0.3616 | 0.923 | 5.67E-06 | 240.6165 | 0.382771 |
| B-NGF | rs61945410 | T | C | -0.9189 | 0.2077 | 0.2726 | 9.67E-06 | 195.3374 | 0.334862 |
| B-NGF | rs74032316 | C | T | 0.8328 | 0.1839 | 0.2434 | 5.95E-06 | 133.117 | 0.255446 |
| B-NGF | rs75426794 | G | A | 0.9351 | 0.2034 | 0.1651 | 4.26E-06 | 123.2403 | 0.241061 |
| B-NGF | rs893507 | C | T | 0.9785 | 0.2204 | 0.1482 | 9.03E-06 | 123.6937 | 0.241734 |
| B-NGF | rs9498000 | A | G | 1.334 | 0.2978 | 0.06684 | 7.45E-06 | 110.7085 | 0.22199 |
| B-NGF | rs966556 | T | C | -0.7344 | 0.1648 | 0.6632 | 8.31E-06 | 123.1597 | 0.240942 |
| CTACK | rs10928931 | C | G | 1.641 | 0.3616 | 0.923 | 5.67E-06 | 240.6165 | 0.382771 |
| CTACK | rs61945410 | T | C | -0.9189 | 0.2077 | 0.2726 | 9.67E-06 | 195.3374 | 0.334862 |
| CTACK | rs74032316 | C | T | 0.8328 | 0.1839 | 0.2434 | 5.95E-06 | 133.117 | 0.255446 |
| CTACK | rs75426794 | G | A | 0.9351 | 0.2034 | 0.1651 | 4.26E-06 | 123.2403 | 0.241061 |
| CTACK | rs893507 | C | T | 0.9785 | 0.2204 | 0.1482 | 9.03E-06 | 123.6937 | 0.241734 |
| CTACK | rs9498000 | A | G | 1.334 | 0.2978 | 0.06684 | 7.45E-06 | 110.7085 | 0.22199 |
| CTACK | rs966556 | T | C | -0.7344 | 0.1648 | 0.6632 | 8.31E-06 | 123.1597 | 0.240942 |
| EOTAXIN | rs10928931 | C | G | 1.641 | 0.3616 | 0.923 | 5.67E-06 | 240.6165 | 0.382771 |
| EOTAXIN | rs61945410 | T | C | -0.9189 | 0.2077 | 0.2726 | 9.67E-06 | 195.3374 | 0.334862 |
| EOTAXIN | rs74032316 | C | T | 0.8328 | 0.1839 | 0.2434 | 5.95E-06 | 133.117 | 0.255446 |
| EOTAXIN | rs75426794 | G | A | 0.9351 | 0.2034 | 0.1651 | 4.26E-06 | 123.2403 | 0.241061 |
| EOTAXIN | rs893507 | C | T | 0.9785 | 0.2204 | 0.1482 | 9.03E-06 | 123.6937 | 0.241734 |
| EOTAXIN | rs9498000 | A | G | 1.334 | 0.2978 | 0.06684 | 7.45E-06 | 110.7085 | 0.22199 |
| EOTAXIN | rs966556 | T | C | -0.7344 | 0.1648 | 0.6632 | 8.31E-06 | 123.1597 | 0.240942 |
| FGF-BASIC | rs10928931 | C | G | 1.641 | 0.3616 | 0.923 | 5.67E-06 | 240.6165 | 0.382771 |
| FGF-BASIC | rs61945410 | T | C | -0.9189 | 0.2077 | 0.2726 | 9.67E-06 | 195.3374 | 0.334862 |
| FGF-BASIC | rs74032316 | C | T | 0.8328 | 0.1839 | 0.2434 | 5.95E-06 | 133.117 | 0.255446 |
| FGF-BASIC | rs75426794 | G | A | 0.9351 | 0.2034 | 0.1651 | 4.26E-06 | 123.2403 | 0.241061 |
| FGF-BASIC | rs893507 | C | T | 0.9785 | 0.2204 | 0.1482 | 9.03E-06 | 123.6937 | 0.241734 |
| FGF-BASIC | rs9498000 | A | G | 1.334 | 0.2978 | 0.06684 | 7.45E-06 | 110.7085 | 0.22199 |
| FGF-BASIC | rs966556 | T | C | -0.7344 | 0.1648 | 0.6632 | 8.31E-06 | 123.1597 | 0.240942 |
| G-CSF | rs10928931 | C | G | 1.641 | 0.3616 | 0.923 | 5.67E-06 | 240.6165 | 0.382771 |
| G-CSF | rs61945410 | T | C | -0.9189 | 0.2077 | 0.2726 | 9.67E-06 | 195.3374 | 0.334862 |
| G-CSF | rs74032316 | C | T | 0.8328 | 0.1839 | 0.2434 | 5.95E-06 | 133.117 | 0.255446 |
| G-CSF | rs75426794 | G | A | 0.9351 | 0.2034 | 0.1651 | 4.26E-06 | 123.2403 | 0.241061 |
| G-CSF | rs893507 | C | T | 0.9785 | 0.2204 | 0.1482 | 9.03E-06 | 123.6937 | 0.241734 |
| G-CSF | rs9498000 | A | G | 1.334 | 0.2978 | 0.06684 | 7.45E-06 | 110.7085 | 0.22199 |
| G-CSF | rs966556 | T | C | -0.7344 | 0.1648 | 0.6632 | 8.31E-06 | 123.1597 | 0.240942 |
| GROA | rs10928931 | C | G | 1.641 | 0.3616 | 0.923 | 5.67E-06 | 240.6165 | 0.382771 |
| GROA | rs61945410 | T | C | -0.9189 | 0.2077 | 0.2726 | 9.67E-06 | 195.3374 | 0.334862 |
| GROA | rs74032316 | C | T | 0.8328 | 0.1839 | 0.2434 | 5.95E-06 | 133.117 | 0.255446 |
| GROA | rs75426794 | G | A | 0.9351 | 0.2034 | 0.1651 | 4.26E-06 | 123.2403 | 0.241061 |
| GROA | rs893507 | C | T | 0.9785 | 0.2204 | 0.1482 | 9.03E-06 | 123.6937 | 0.241734 |
| GROA | rs9498000 | A | G | 1.334 | 0.2978 | 0.06684 | 7.45E-06 | 110.7085 | 0.22199 |
| GROA | rs966556 | T | C | -0.7344 | 0.1648 | 0.6632 | 8.31E-06 | 123.1597 | 0.240942 |
| HGF | rs10928931 | C | G | 1.641 | 0.3616 | 0.923 | 5.67E-06 | 240.6165 | 0.382771 |
| HGF | rs61945410 | T | C | -0.9189 | 0.2077 | 0.2726 | 9.67E-06 | 195.3374 | 0.334862 |
| HGF | rs74032316 | C | T | 0.8328 | 0.1839 | 0.2434 | 5.95E-06 | 133.117 | 0.255446 |
| HGF | rs75426794 | G | A | 0.9351 | 0.2034 | 0.1651 | 4.26E-06 | 123.2403 | 0.241061 |
| HGF | rs893507 | C | T | 0.9785 | 0.2204 | 0.1482 | 9.03E-06 | 123.6937 | 0.241734 |
| HGF | rs9498000 | A | G | 1.334 | 0.2978 | 0.06684 | 7.45E-06 | 110.7085 | 0.22199 |
| HGF | rs966556 | T | C | -0.7344 | 0.1648 | 0.6632 | 8.31E-06 | 123.1597 | 0.240942 |
| IFN-G | rs10928931 | C | G | 1.641 | 0.3616 | 0.923 | 5.67E-06 | 240.6165 | 0.382771 |
| IFN-G | rs61945410 | T | C | -0.9189 | 0.2077 | 0.2726 | 9.67E-06 | 195.3374 | 0.334862 |
| IFN-G | rs74032316 | C | T | 0.8328 | 0.1839 | 0.2434 | 5.95E-06 | 133.117 | 0.255446 |
| IFN-G | rs75426794 | G | A | 0.9351 | 0.2034 | 0.1651 | 4.26E-06 | 123.2403 | 0.241061 |
| IFN-G | rs893507 | C | T | 0.9785 | 0.2204 | 0.1482 | 9.03E-06 | 123.6937 | 0.241734 |
| IFN-G | rs9498000 | A | G | 1.334 | 0.2978 | 0.06684 | 7.45E-06 | 110.7085 | 0.22199 |
| IFN-G | rs966556 | T | C | -0.7344 | 0.1648 | 0.6632 | 8.31E-06 | 123.1597 | 0.240942 |
| IL-10 | rs10928931 | C | G | 1.641 | 0.3616 | 0.923 | 5.67E-06 | 240.6165 | 0.382771 |
| IL-10 | rs61945410 | T | C | -0.9189 | 0.2077 | 0.2726 | 9.67E-06 | 195.3374 | 0.334862 |
| IL-10 | rs74032316 | C | T | 0.8328 | 0.1839 | 0.2434 | 5.95E-06 | 133.117 | 0.255446 |
| IL-10 | rs75426794 | G | A | 0.9351 | 0.2034 | 0.1651 | 4.26E-06 | 123.2403 | 0.241061 |
| IL-10 | rs893507 | C | T | 0.9785 | 0.2204 | 0.1482 | 9.03E-06 | 123.6937 | 0.241734 |
| IL-10 | rs9498000 | A | G | 1.334 | 0.2978 | 0.06684 | 7.45E-06 | 110.7085 | 0.22199 |
| IL-10 | rs966556 | T | C | -0.7344 | 0.1648 | 0.6632 | 8.31E-06 | 123.1597 | 0.240942 |
| IL-12 | rs10928931 | C | G | 1.641 | 0.3616 | 0.923 | 5.67E-06 | 240.6165 | 0.382771 |
| IL-12 | rs61945410 | T | C | -0.9189 | 0.2077 | 0.2726 | 9.67E-06 | 195.3374 | 0.334862 |
| IL-12 | rs74032316 | C | T | 0.8328 | 0.1839 | 0.2434 | 5.95E-06 | 133.117 | 0.255446 |
| IL-12 | rs75426794 | G | A | 0.9351 | 0.2034 | 0.1651 | 4.26E-06 | 123.2403 | 0.241061 |
| IL-12 | rs893507 | C | T | 0.9785 | 0.2204 | 0.1482 | 9.03E-06 | 123.6937 | 0.241734 |
| IL-12 | rs9498000 | A | G | 1.334 | 0.2978 | 0.06684 | 7.45E-06 | 110.7085 | 0.22199 |
| IL-12 | rs966556 | T | C | -0.7344 | 0.1648 | 0.6632 | 8.31E-06 | 123.1597 | 0.240942 |
| IL-13 | rs10928931 | C | G | 1.641 | 0.3616 | 0.923 | 5.67E-06 | 240.6165 | 0.382771 |
| IL-13 | rs61945410 | T | C | -0.9189 | 0.2077 | 0.2726 | 9.67E-06 | 195.3374 | 0.334862 |
| IL-13 | rs74032316 | C | T | 0.8328 | 0.1839 | 0.2434 | 5.95E-06 | 133.117 | 0.255446 |
| IL-13 | rs75426794 | G | A | 0.9351 | 0.2034 | 0.1651 | 4.26E-06 | 123.2403 | 0.241061 |
| IL-13 | rs893507 | C | T | 0.9785 | 0.2204 | 0.1482 | 9.03E-06 | 123.6937 | 0.241734 |
| IL-13 | rs9498000 | A | G | 1.334 | 0.2978 | 0.06684 | 7.45E-06 | 110.7085 | 0.22199 |
| IL-13 | rs966556 | T | C | -0.7344 | 0.1648 | 0.6632 | 8.31E-06 | 123.1597 | 0.240942 |
| IL-16 | rs10928931 | C | G | 1.641 | 0.3616 | 0.923 | 5.67E-06 | 240.6165 | 0.382771 |
| IL-16 | rs61945410 | T | C | -0.9189 | 0.2077 | 0.2726 | 9.67E-06 | 195.3374 | 0.334862 |
| IL-16 | rs74032316 | C | T | 0.8328 | 0.1839 | 0.2434 | 5.95E-06 | 133.117 | 0.255446 |
| IL-16 | rs75426794 | G | A | 0.9351 | 0.2034 | 0.1651 | 4.26E-06 | 123.2403 | 0.241061 |
| IL-16 | rs893507 | C | T | 0.9785 | 0.2204 | 0.1482 | 9.03E-06 | 123.6937 | 0.241734 |
| IL-16 | rs9498000 | A | G | 1.334 | 0.2978 | 0.06684 | 7.45E-06 | 110.7085 | 0.22199 |
| IL-16 | rs966556 | T | C | -0.7344 | 0.1648 | 0.6632 | 8.31E-06 | 123.1597 | 0.240942 |
| IL-17 | rs10928931 | C | G | 1.641 | 0.3616 | 0.923 | 5.67E-06 | 240.6165 | 0.382771 |
| IL-17 | rs61945410 | T | C | -0.9189 | 0.2077 | 0.2726 | 9.67E-06 | 195.3374 | 0.334862 |
| IL-17 | rs74032316 | C | T | 0.8328 | 0.1839 | 0.2434 | 5.95E-06 | 133.117 | 0.255446 |
| IL-17 | rs75426794 | G | A | 0.9351 | 0.2034 | 0.1651 | 4.26E-06 | 123.2403 | 0.241061 |
| IL-17 | rs893507 | C | T | 0.9785 | 0.2204 | 0.1482 | 9.03E-06 | 123.6937 | 0.241734 |
| IL-17 | rs9498000 | A | G | 1.334 | 0.2978 | 0.06684 | 7.45E-06 | 110.7085 | 0.22199 |
| IL-17 | rs966556 | T | C | -0.7344 | 0.1648 | 0.6632 | 8.31E-06 | 123.1597 | 0.240942 |
| IL-18 | rs10928931 | C | G | 1.641 | 0.3616 | 0.923 | 5.67E-06 | 240.6165 | 0.382771 |
| IL-18 | rs61945410 | T | C | -0.9189 | 0.2077 | 0.2726 | 9.67E-06 | 195.3374 | 0.334862 |
| IL-18 | rs74032316 | C | T | 0.8328 | 0.1839 | 0.2434 | 5.95E-06 | 133.117 | 0.255446 |
| IL-18 | rs75426794 | G | A | 0.9351 | 0.2034 | 0.1651 | 4.26E-06 | 123.2403 | 0.241061 |
| IL-18 | rs893507 | C | T | 0.9785 | 0.2204 | 0.1482 | 9.03E-06 | 123.6937 | 0.241734 |
| IL-18 | rs9498000 | A | G | 1.334 | 0.2978 | 0.06684 | 7.45E-06 | 110.7085 | 0.22199 |
| IL-18 | rs966556 | T | C | -0.7344 | 0.1648 | 0.6632 | 8.31E-06 | 123.1597 | 0.240942 |
| IL-1B | rs10928931 | C | G | 1.641 | 0.3616 | 0.923 | 5.67E-06 | 240.6165 | 0.382771 |
| IL-1B | rs61945410 | T | C | -0.9189 | 0.2077 | 0.2726 | 9.67E-06 | 195.3374 | 0.334862 |
| IL-1B | rs74032316 | C | T | 0.8328 | 0.1839 | 0.2434 | 5.95E-06 | 133.117 | 0.255446 |
| IL-1B | rs75426794 | G | A | 0.9351 | 0.2034 | 0.1651 | 4.26E-06 | 123.2403 | 0.241061 |
| IL-1B | rs893507 | C | T | 0.9785 | 0.2204 | 0.1482 | 9.03E-06 | 123.6937 | 0.241734 |
| IL-1B | rs9498000 | A | G | 1.334 | 0.2978 | 0.06684 | 7.45E-06 | 110.7085 | 0.22199 |
| IL-1B | rs966556 | T | C | -0.7344 | 0.1648 | 0.6632 | 8.31E-06 | 123.1597 | 0.240942 |
| IL-1RA | rs10928931 | C | G | 1.641 | 0.3616 | 0.923 | 5.67E-06 | 240.6165 | 0.382771 |
| IL-1RA | rs61945410 | T | C | -0.9189 | 0.2077 | 0.2726 | 9.67E-06 | 195.3374 | 0.334862 |
| IL-1RA | rs74032316 | C | T | 0.8328 | 0.1839 | 0.2434 | 5.95E-06 | 133.117 | 0.255446 |
| IL-1RA | rs75426794 | G | A | 0.9351 | 0.2034 | 0.1651 | 4.26E-06 | 123.2403 | 0.241061 |
| IL-1RA | rs893507 | C | T | 0.9785 | 0.2204 | 0.1482 | 9.03E-06 | 123.6937 | 0.241734 |
| IL-1RA | rs9498000 | A | G | 1.334 | 0.2978 | 0.06684 | 7.45E-06 | 110.7085 | 0.22199 |
| IL-1RA | rs966556 | T | C | -0.7344 | 0.1648 | 0.6632 | 8.31E-06 | 123.1597 | 0.240942 |
| IL-2 | rs10928931 | C | G | 1.641 | 0.3616 | 0.923 | 5.67E-06 | 240.6165 | 0.382771 |
| IL-2 | rs61945410 | T | C | -0.9189 | 0.2077 | 0.2726 | 9.67E-06 | 195.3374 | 0.334862 |
| IL-2 | rs74032316 | C | T | 0.8328 | 0.1839 | 0.2434 | 5.95E-06 | 133.117 | 0.255446 |
| IL-2 | rs75426794 | G | A | 0.9351 | 0.2034 | 0.1651 | 4.26E-06 | 123.2403 | 0.241061 |
| IL-2 | rs893507 | C | T | 0.9785 | 0.2204 | 0.1482 | 9.03E-06 | 123.6937 | 0.241734 |
| IL-2 | rs9498000 | A | G | 1.334 | 0.2978 | 0.06684 | 7.45E-06 | 110.7085 | 0.22199 |
| IL-2 | rs966556 | T | C | -0.7344 | 0.1648 | 0.6632 | 8.31E-06 | 123.1597 | 0.240942 |
| IL-2RA | rs10928931 | C | G | 1.641 | 0.3616 | 0.923 | 5.67E-06 | 240.6165 | 0.382771 |
| IL-2RA | rs61945410 | T | C | -0.9189 | 0.2077 | 0.2726 | 9.67E-06 | 195.3374 | 0.334862 |
| IL-2RA | rs74032316 | C | T | 0.8328 | 0.1839 | 0.2434 | 5.95E-06 | 133.117 | 0.255446 |
| IL-2RA | rs75426794 | G | A | 0.9351 | 0.2034 | 0.1651 | 4.26E-06 | 123.2403 | 0.241061 |
| IL-2RA | rs893507 | C | T | 0.9785 | 0.2204 | 0.1482 | 9.03E-06 | 123.6937 | 0.241734 |
| IL-2RA | rs9498000 | A | G | 1.334 | 0.2978 | 0.06684 | 7.45E-06 | 110.7085 | 0.22199 |
| IL-2RA | rs966556 | T | C | -0.7344 | 0.1648 | 0.6632 | 8.31E-06 | 123.1597 | 0.240942 |
| IL-4 | rs10928931 | C | G | 1.641 | 0.3616 | 0.923 | 5.67E-06 | 240.6165 | 0.382771 |
| IL-4 | rs61945410 | T | C | -0.9189 | 0.2077 | 0.2726 | 9.67E-06 | 195.3374 | 0.334862 |
| IL-4 | rs74032316 | C | T | 0.8328 | 0.1839 | 0.2434 | 5.95E-06 | 133.117 | 0.255446 |
| IL-4 | rs75426794 | G | A | 0.9351 | 0.2034 | 0.1651 | 4.26E-06 | 123.2403 | 0.241061 |
| IL-4 | rs893507 | C | T | 0.9785 | 0.2204 | 0.1482 | 9.03E-06 | 123.6937 | 0.241734 |
| IL-4 | rs9498000 | A | G | 1.334 | 0.2978 | 0.06684 | 7.45E-06 | 110.7085 | 0.22199 |
| IL-4 | rs966556 | T | C | -0.7344 | 0.1648 | 0.6632 | 8.31E-06 | 123.1597 | 0.240942 |
| IL-5 | rs10928931 | C | G | 1.641 | 0.3616 | 0.923 | 5.67E-06 | 240.6165 | 0.382771 |
| IL-5 | rs61945410 | T | C | -0.9189 | 0.2077 | 0.2726 | 9.67E-06 | 195.3374 | 0.334862 |
| IL-5 | rs74032316 | C | T | 0.8328 | 0.1839 | 0.2434 | 5.95E-06 | 133.117 | 0.255446 |
| IL-5 | rs75426794 | G | A | 0.9351 | 0.2034 | 0.1651 | 4.26E-06 | 123.2403 | 0.241061 |
| IL-5 | rs893507 | C | T | 0.9785 | 0.2204 | 0.1482 | 9.03E-06 | 123.6937 | 0.241734 |
| IL-5 | rs9498000 | A | G | 1.334 | 0.2978 | 0.06684 | 7.45E-06 | 110.7085 | 0.22199 |
| IL-5 | rs966556 | T | C | -0.7344 | 0.1648 | 0.6632 | 8.31E-06 | 123.1597 | 0.240942 |
| IL-6 | rs10928931 | C | G | 1.641 | 0.3616 | 0.923 | 5.67E-06 | 240.6165 | 0.382771 |
| IL-6 | rs61945410 | T | C | -0.9189 | 0.2077 | 0.2726 | 9.67E-06 | 195.3374 | 0.334862 |
| IL-6 | rs74032316 | C | T | 0.8328 | 0.1839 | 0.2434 | 5.95E-06 | 133.117 | 0.255446 |
| IL-6 | rs75426794 | G | A | 0.9351 | 0.2034 | 0.1651 | 4.26E-06 | 123.2403 | 0.241061 |
| IL-6 | rs893507 | C | T | 0.9785 | 0.2204 | 0.1482 | 9.03E-06 | 123.6937 | 0.241734 |
| IL-6 | rs9498000 | A | G | 1.334 | 0.2978 | 0.06684 | 7.45E-06 | 110.7085 | 0.22199 |
| IL-6 | rs966556 | T | C | -0.7344 | 0.1648 | 0.6632 | 8.31E-06 | 123.1597 | 0.240942 |
| IL-7 | rs10928931 | C | G | 1.641 | 0.3616 | 0.923 | 5.67E-06 | 240.6165 | 0.382771 |
| IL-7 | rs61945410 | T | C | -0.9189 | 0.2077 | 0.2726 | 9.67E-06 | 195.3374 | 0.334862 |
| IL-7 | rs74032316 | C | T | 0.8328 | 0.1839 | 0.2434 | 5.95E-06 | 133.117 | 0.255446 |
| IL-7 | rs75426794 | G | A | 0.9351 | 0.2034 | 0.1651 | 4.26E-06 | 123.2403 | 0.241061 |
| IL-7 | rs893507 | C | T | 0.9785 | 0.2204 | 0.1482 | 9.03E-06 | 123.6937 | 0.241734 |
| IL-7 | rs9498000 | A | G | 1.334 | 0.2978 | 0.06684 | 7.45E-06 | 110.7085 | 0.22199 |
| IL-7 | rs966556 | T | C | -0.7344 | 0.1648 | 0.6632 | 8.31E-06 | 123.1597 | 0.240942 |
| IL-8 | rs10928931 | C | G | 1.641 | 0.3616 | 0.923 | 5.67E-06 | 240.6165 | 0.382771 |
| IL-8 | rs61945410 | T | C | -0.9189 | 0.2077 | 0.2726 | 9.67E-06 | 195.3374 | 0.334862 |
| IL-8 | rs74032316 | C | T | 0.8328 | 0.1839 | 0.2434 | 5.95E-06 | 133.117 | 0.255446 |
| IL-8 | rs75426794 | G | A | 0.9351 | 0.2034 | 0.1651 | 4.26E-06 | 123.2403 | 0.241061 |
| IL-8 | rs893507 | C | T | 0.9785 | 0.2204 | 0.1482 | 9.03E-06 | 123.6937 | 0.241734 |
| IL-8 | rs9498000 | A | G | 1.334 | 0.2978 | 0.06684 | 7.45E-06 | 110.7085 | 0.22199 |
| IL-8 | rs966556 | T | C | -0.7344 | 0.1648 | 0.6632 | 8.31E-06 | 123.1597 | 0.240942 |
| IL-9 | rs10928931 | C | G | 1.641 | 0.3616 | 0.923 | 5.67E-06 | 240.6165 | 0.382771 |
| IL-9 | rs61945410 | T | C | -0.9189 | 0.2077 | 0.2726 | 9.67E-06 | 195.3374 | 0.334862 |
| IL-9 | rs74032316 | C | T | 0.8328 | 0.1839 | 0.2434 | 5.95E-06 | 133.117 | 0.255446 |
| IL-9 | rs75426794 | G | A | 0.9351 | 0.2034 | 0.1651 | 4.26E-06 | 123.2403 | 0.241061 |
| IL-9 | rs893507 | C | T | 0.9785 | 0.2204 | 0.1482 | 9.03E-06 | 123.6937 | 0.241734 |
| IL-9 | rs9498000 | A | G | 1.334 | 0.2978 | 0.06684 | 7.45E-06 | 110.7085 | 0.22199 |
| IL-9 | rs966556 | T | C | -0.7344 | 0.1648 | 0.6632 | 8.31E-06 | 123.1597 | 0.240942 |
| IP-10 | rs10928931 | C | G | 1.641 | 0.3616 | 0.923 | 5.67E-06 | 240.6165 | 0.382771 |
| IP-10 | rs61945410 | T | C | -0.9189 | 0.2077 | 0.2726 | 9.67E-06 | 195.3374 | 0.334862 |
| IP-10 | rs74032316 | C | T | 0.8328 | 0.1839 | 0.2434 | 5.95E-06 | 133.117 | 0.255446 |
| IP-10 | rs75426794 | G | A | 0.9351 | 0.2034 | 0.1651 | 4.26E-06 | 123.2403 | 0.241061 |
| IP-10 | rs893507 | C | T | 0.9785 | 0.2204 | 0.1482 | 9.03E-06 | 123.6937 | 0.241734 |
| IP-10 | rs9498000 | A | G | 1.334 | 0.2978 | 0.06684 | 7.45E-06 | 110.7085 | 0.22199 |
| IP-10 | rs966556 | T | C | -0.7344 | 0.1648 | 0.6632 | 8.31E-06 | 123.1597 | 0.240942 |
| M-CSF | rs10928931 | C | G | 1.641 | 0.3616 | 0.923 | 5.67E-06 | 240.6165 | 0.382771 |
| M-CSF | rs61945410 | T | C | -0.9189 | 0.2077 | 0.2726 | 9.67E-06 | 195.3374 | 0.334862 |
| M-CSF | rs74032316 | C | T | 0.8328 | 0.1839 | 0.2434 | 5.95E-06 | 133.117 | 0.255446 |
| M-CSF | rs75426794 | G | A | 0.9351 | 0.2034 | 0.1651 | 4.26E-06 | 123.2403 | 0.241061 |
| M-CSF | rs893507 | C | T | 0.9785 | 0.2204 | 0.1482 | 9.03E-06 | 123.6937 | 0.241734 |
| M-CSF | rs9498000 | A | G | 1.334 | 0.2978 | 0.06684 | 7.45E-06 | 110.7085 | 0.22199 |
| M-CSF | rs966556 | T | C | -0.7344 | 0.1648 | 0.6632 | 8.31E-06 | 123.1597 | 0.240942 |
| MCP-1 | rs10928931 | C | G | 1.641 | 0.3616 | 0.923 | 5.67E-06 | 240.6165 | 0.382771 |
| MCP-1 | rs61945410 | T | C | -0.9189 | 0.2077 | 0.2726 | 9.67E-06 | 195.3374 | 0.334862 |
| MCP-1 | rs74032316 | C | T | 0.8328 | 0.1839 | 0.2434 | 5.95E-06 | 133.117 | 0.255446 |
| MCP-1 | rs75426794 | G | A | 0.9351 | 0.2034 | 0.1651 | 4.26E-06 | 123.2403 | 0.241061 |
| MCP-1 | rs893507 | C | T | 0.9785 | 0.2204 | 0.1482 | 9.03E-06 | 123.6937 | 0.241734 |
| MCP-1 | rs9498000 | A | G | 1.334 | 0.2978 | 0.06684 | 7.45E-06 | 110.7085 | 0.22199 |
| MCP-1 | rs966556 | T | C | -0.7344 | 0.1648 | 0.6632 | 8.31E-06 | 123.1597 | 0.240942 |
| MCP-3 | rs10928931 | C | G | 1.641 | 0.3616 | 0.923 | 5.67E-06 | 240.6165 | 0.382771 |
| MCP-3 | rs61945410 | T | C | -0.9189 | 0.2077 | 0.2726 | 9.67E-06 | 195.3374 | 0.334862 |
| MCP-3 | rs74032316 | C | T | 0.8328 | 0.1839 | 0.2434 | 5.95E-06 | 133.117 | 0.255446 |
| MCP-3 | rs75426794 | G | A | 0.9351 | 0.2034 | 0.1651 | 4.26E-06 | 123.2403 | 0.241061 |
| MCP-3 | rs893507 | C | T | 0.9785 | 0.2204 | 0.1482 | 9.03E-06 | 123.6937 | 0.241734 |
| MCP-3 | rs9498000 | A | G | 1.334 | 0.2978 | 0.06684 | 7.45E-06 | 110.7085 | 0.22199 |
| MCP-3 | rs966556 | T | C | -0.7344 | 0.1648 | 0.6632 | 8.31E-06 | 123.1597 | 0.240942 |
| MIF | rs10928931 | C | G | 1.641 | 0.3616 | 0.923 | 5.67E-06 | 240.6165 | 0.382771 |
| MIF | rs61945410 | T | C | -0.9189 | 0.2077 | 0.2726 | 9.67E-06 | 195.3374 | 0.334862 |
| MIF | rs74032316 | C | T | 0.8328 | 0.1839 | 0.2434 | 5.95E-06 | 133.117 | 0.255446 |
| MIF | rs75426794 | G | A | 0.9351 | 0.2034 | 0.1651 | 4.26E-06 | 123.2403 | 0.241061 |
| MIF | rs893507 | C | T | 0.9785 | 0.2204 | 0.1482 | 9.03E-06 | 123.6937 | 0.241734 |
| MIF | rs9498000 | A | G | 1.334 | 0.2978 | 0.06684 | 7.45E-06 | 110.7085 | 0.22199 |
| MIF | rs966556 | T | C | -0.7344 | 0.1648 | 0.6632 | 8.31E-06 | 123.1597 | 0.240942 |
| MIG | rs10928931 | C | G | 1.641 | 0.3616 | 0.923 | 5.67E-06 | 240.6165 | 0.382771 |
| MIG | rs61945410 | T | C | -0.9189 | 0.2077 | 0.2726 | 9.67E-06 | 195.3374 | 0.334862 |
| MIG | rs74032316 | C | T | 0.8328 | 0.1839 | 0.2434 | 5.95E-06 | 133.117 | 0.255446 |
| MIG | rs75426794 | G | A | 0.9351 | 0.2034 | 0.1651 | 4.26E-06 | 123.2403 | 0.241061 |
| MIG | rs893507 | C | T | 0.9785 | 0.2204 | 0.1482 | 9.03E-06 | 123.6937 | 0.241734 |
| MIG | rs9498000 | A | G | 1.334 | 0.2978 | 0.06684 | 7.45E-06 | 110.7085 | 0.22199 |
| MIG | rs966556 | T | C | -0.7344 | 0.1648 | 0.6632 | 8.31E-06 | 123.1597 | 0.240942 |
| MIP-1A | rs10928931 | C | G | 1.641 | 0.3616 | 0.923 | 5.67E-06 | 240.6165 | 0.382771 |
| MIP-1A | rs61945410 | T | C | -0.9189 | 0.2077 | 0.2726 | 9.67E-06 | 195.3374 | 0.334862 |
| MIP-1A | rs74032316 | C | T | 0.8328 | 0.1839 | 0.2434 | 5.95E-06 | 133.117 | 0.255446 |
| MIP-1A | rs75426794 | G | A | 0.9351 | 0.2034 | 0.1651 | 4.26E-06 | 123.2403 | 0.241061 |
| MIP-1A | rs893507 | C | T | 0.9785 | 0.2204 | 0.1482 | 9.03E-06 | 123.6937 | 0.241734 |
| MIP-1A | rs9498000 | A | G | 1.334 | 0.2978 | 0.06684 | 7.45E-06 | 110.7085 | 0.22199 |
| MIP-1A | rs966556 | T | C | -0.7344 | 0.1648 | 0.6632 | 8.31E-06 | 123.1597 | 0.240942 |
| MIP-1B | rs10928931 | C | G | 1.641 | 0.3616 | 0.923 | 5.67E-06 | 240.6165 | 0.382771 |
| MIP-1B | rs61945410 | T | C | -0.9189 | 0.2077 | 0.2726 | 9.67E-06 | 195.3374 | 0.334862 |
| MIP-1B | rs74032316 | C | T | 0.8328 | 0.1839 | 0.2434 | 5.95E-06 | 133.117 | 0.255446 |
| MIP-1B | rs75426794 | G | A | 0.9351 | 0.2034 | 0.1651 | 4.26E-06 | 123.2403 | 0.241061 |
| MIP-1B | rs893507 | C | T | 0.9785 | 0.2204 | 0.1482 | 9.03E-06 | 123.6937 | 0.241734 |
| MIP-1B | rs9498000 | A | G | 1.334 | 0.2978 | 0.06684 | 7.45E-06 | 110.7085 | 0.22199 |
| MIP-1B | rs966556 | T | C | -0.7344 | 0.1648 | 0.6632 | 8.31E-06 | 123.1597 | 0.240942 |
| PDGF-BB | rs10928931 | C | G | 1.641 | 0.3616 | 0.923 | 5.67E-06 | 240.6165 | 0.382771 |
| PDGF-BB | rs61945410 | T | C | -0.9189 | 0.2077 | 0.2726 | 9.67E-06 | 195.3374 | 0.334862 |
| PDGF-BB | rs74032316 | C | T | 0.8328 | 0.1839 | 0.2434 | 5.95E-06 | 133.117 | 0.255446 |
| PDGF-BB | rs75426794 | G | A | 0.9351 | 0.2034 | 0.1651 | 4.26E-06 | 123.2403 | 0.241061 |
| PDGF-BB | rs893507 | C | T | 0.9785 | 0.2204 | 0.1482 | 9.03E-06 | 123.6937 | 0.241734 |
| PDGF-BB | rs9498000 | A | G | 1.334 | 0.2978 | 0.06684 | 7.45E-06 | 110.7085 | 0.22199 |
| PDGF-BB | rs966556 | T | C | -0.7344 | 0.1648 | 0.6632 | 8.31E-06 | 123.1597 | 0.240942 |
| RANTES | rs10928931 | C | G | 1.641 | 0.3616 | 0.923 | 5.67E-06 | 240.6165 | 0.382771 |
| RANTES | rs61945410 | T | C | -0.9189 | 0.2077 | 0.2726 | 9.67E-06 | 195.3374 | 0.334862 |
| RANTES | rs74032316 | C | T | 0.8328 | 0.1839 | 0.2434 | 5.95E-06 | 133.117 | 0.255446 |
| RANTES | rs75426794 | G | A | 0.9351 | 0.2034 | 0.1651 | 4.26E-06 | 123.2403 | 0.241061 |
| RANTES | rs893507 | C | T | 0.9785 | 0.2204 | 0.1482 | 9.03E-06 | 123.6937 | 0.241734 |
| RANTES | rs9498000 | A | G | 1.334 | 0.2978 | 0.06684 | 7.45E-06 | 110.7085 | 0.22199 |
| RANTES | rs966556 | T | C | -0.7344 | 0.1648 | 0.6632 | 8.31E-06 | 123.1597 | 0.240942 |
| SCF | rs10928931 | C | G | 1.641 | 0.3616 | 0.923 | 5.67E-06 | 240.6165 | 0.382771 |
| SCF | rs61945410 | T | C | -0.9189 | 0.2077 | 0.2726 | 9.67E-06 | 195.3374 | 0.334862 |
| SCF | rs74032316 | C | T | 0.8328 | 0.1839 | 0.2434 | 5.95E-06 | 133.117 | 0.255446 |
| SCF | rs75426794 | G | A | 0.9351 | 0.2034 | 0.1651 | 4.26E-06 | 123.2403 | 0.241061 |
| SCF | rs893507 | C | T | 0.9785 | 0.2204 | 0.1482 | 9.03E-06 | 123.6937 | 0.241734 |
| SCF | rs9498000 | A | G | 1.334 | 0.2978 | 0.06684 | 7.45E-06 | 110.7085 | 0.22199 |
| SCF | rs966556 | T | C | -0.7344 | 0.1648 | 0.6632 | 8.31E-06 | 123.1597 | 0.240942 |
| SCGF-B | rs10928931 | C | G | 1.641 | 0.3616 | 0.923 | 5.67E-06 | 240.6165 | 0.382771 |
| SCGF-B | rs61945410 | T | C | -0.9189 | 0.2077 | 0.2726 | 9.67E-06 | 195.3374 | 0.334862 |
| SCGF-B | rs74032316 | C | T | 0.8328 | 0.1839 | 0.2434 | 5.95E-06 | 133.117 | 0.255446 |
| SCGF-B | rs75426794 | G | A | 0.9351 | 0.2034 | 0.1651 | 4.26E-06 | 123.2403 | 0.241061 |
| SCGF-B | rs893507 | C | T | 0.9785 | 0.2204 | 0.1482 | 9.03E-06 | 123.6937 | 0.241734 |
| SCGF-B | rs9498000 | A | G | 1.334 | 0.2978 | 0.06684 | 7.45E-06 | 110.7085 | 0.22199 |
| SCGF-B | rs966556 | T | C | -0.7344 | 0.1648 | 0.6632 | 8.31E-06 | 123.1597 | 0.240942 |
| SDF-1A | rs10928931 | C | G | 1.641 | 0.3616 | 0.923 | 5.67E-06 | 240.6165 | 0.382771 |
| SDF-1A | rs61945410 | T | C | -0.9189 | 0.2077 | 0.2726 | 9.67E-06 | 195.3374 | 0.334862 |
| SDF-1A | rs74032316 | C | T | 0.8328 | 0.1839 | 0.2434 | 5.95E-06 | 133.117 | 0.255446 |
| SDF-1A | rs75426794 | G | A | 0.9351 | 0.2034 | 0.1651 | 4.26E-06 | 123.2403 | 0.241061 |
| SDF-1A | rs893507 | C | T | 0.9785 | 0.2204 | 0.1482 | 9.03E-06 | 123.6937 | 0.241734 |
| SDF-1A | rs9498000 | A | G | 1.334 | 0.2978 | 0.06684 | 7.45E-06 | 110.7085 | 0.22199 |
| SDF-1A | rs966556 | T | C | -0.7344 | 0.1648 | 0.6632 | 8.31E-06 | 123.1597 | 0.240942 |
| TNF-A | rs10928931 | C | G | 1.641 | 0.3616 | 0.923 | 5.67E-06 | 240.6165 | 0.382771 |
| TNF-A | rs61945410 | T | C | -0.9189 | 0.2077 | 0.2726 | 9.67E-06 | 195.3374 | 0.334862 |
| TNF-A | rs74032316 | C | T | 0.8328 | 0.1839 | 0.2434 | 5.95E-06 | 133.117 | 0.255446 |
| TNF-A | rs75426794 | G | A | 0.9351 | 0.2034 | 0.1651 | 4.26E-06 | 123.2403 | 0.241061 |
| TNF-A | rs893507 | C | T | 0.9785 | 0.2204 | 0.1482 | 9.03E-06 | 123.6937 | 0.241734 |
| TNF-A | rs9498000 | A | G | 1.334 | 0.2978 | 0.06684 | 7.45E-06 | 110.7085 | 0.22199 |
| TNF-A | rs966556 | T | C | -0.7344 | 0.1648 | 0.6632 | 8.31E-06 | 123.1597 | 0.240942 |
| TNF-B | rs10928931 | C | G | 1.641 | 0.3616 | 0.923 | 5.67E-06 | 240.6165 | 0.382771 |
| TNF-B | rs61945410 | T | C | -0.9189 | 0.2077 | 0.2726 | 9.67E-06 | 195.3374 | 0.334862 |
| TNF-B | rs74032316 | C | T | 0.8328 | 0.1839 | 0.2434 | 5.95E-06 | 133.117 | 0.255446 |
| TNF-B | rs75426794 | G | A | 0.9351 | 0.2034 | 0.1651 | 4.26E-06 | 123.2403 | 0.241061 |
| TNF-B | rs893507 | C | T | 0.9785 | 0.2204 | 0.1482 | 9.03E-06 | 123.6937 | 0.241734 |
| TNF-B | rs9498000 | A | G | 1.334 | 0.2978 | 0.06684 | 7.45E-06 | 110.7085 | 0.22199 |
| TNF-B | rs966556 | T | C | -0.7344 | 0.1648 | 0.6632 | 8.31E-06 | 123.1597 | 0.240942 |
| TRAIL | rs10928931 | C | G | 1.641 | 0.3616 | 0.923 | 5.67E-06 | 240.6165 | 0.382771 |
| TRAIL | rs61945410 | T | C | -0.9189 | 0.2077 | 0.2726 | 9.67E-06 | 195.3374 | 0.334862 |
| TRAIL | rs74032316 | C | T | 0.8328 | 0.1839 | 0.2434 | 5.95E-06 | 133.117 | 0.255446 |
| TRAIL | rs75426794 | G | A | 0.9351 | 0.2034 | 0.1651 | 4.26E-06 | 123.2403 | 0.241061 |
| TRAIL | rs893507 | C | T | 0.9785 | 0.2204 | 0.1482 | 9.03E-06 | 123.6937 | 0.241734 |
| TRAIL | rs9498000 | A | G | 1.334 | 0.2978 | 0.06684 | 7.45E-06 | 110.7085 | 0.22199 |
| TRAIL | rs966556 | T | C | -0.7344 | 0.1648 | 0.6632 | 8.31E-06 | 123.1597 | 0.240942 |
| VEGF | rs10928931 | C | G | 1.641 | 0.3616 | 0.923 | 5.67E-06 | 240.6165 | 0.382771 |
| VEGF | rs61945410 | T | C | -0.9189 | 0.2077 | 0.2726 | 9.67E-06 | 195.3374 | 0.334862 |
| VEGF | rs74032316 | C | T | 0.8328 | 0.1839 | 0.2434 | 5.95E-06 | 133.117 | 0.255446 |
| VEGF | rs75426794 | G | A | 0.9351 | 0.2034 | 0.1651 | 4.26E-06 | 123.2403 | 0.241061 |
| VEGF | rs893507 | C | T | 0.9785 | 0.2204 | 0.1482 | 9.03E-06 | 123.6937 | 0.241734 |
| VEGF | rs9498000 | A | G | 1.334 | 0.2978 | 0.06684 | 7.45E-06 | 110.7085 | 0.22199 |
| VEGF | rs966556 | T | C | -0.7344 | 0.1648 | 0.6632 | 8.31E-06 | 123.1597 | 0.240942 |
